# Electron-Donating Ligand in Oxidorhenium(V) Chemistry: Consequences for Isomerism and Catalyst Properties

**DOI:** 10.1021/acs.inorgchem.5c04871

**Published:** 2026-01-20

**Authors:** Cornelia Rom, Christof Holzer, Antoine Dupé, Ferdinand Belaj, Nadia C. Mösch-Zanetti, Jörg A. Schachner

**Affiliations:** † Institute of Chemistry, University of Graz, Schubertstr. 1, 8010 Graz, Austria; ‡ Institute of Quantum Materials and Technologies, Karlsruhe Institute of Technology, Wolfgang-Gaede-Str. 1, 76131 Karlsruhe, Germany

## Abstract

The bidentate, monoanionic dimethyloxazoline-phenol ligand H**L1** was used to synthesize the corresponding oxidorhenium­(V) complex [ReOCl­(**L1**)_2_] (**2**). Ligand H**L1** is equipped with two electron-donating *tert-*butyl substituents. The HdmozR class of ligands has so far enabled the stereoselective synthesis of the so-called *N*,*N*-*trans* isomers of oxidorhenium­(V) complexes. In contrast, when precursor complex [ReOCl_3_(OPPh_3_)­(SMe_2_)] (**P1**) and H**L1** are reacted, in addition to the expected *N*,*N*-*trans* isomer (*trans-* **2**), also the *N*,*N*-*cis* isomer (*cis*-**2**) is formed. So far, this isomerism has only been observed for the nonmethylated phenol-oxazoline ligand Hoz, resulting in mixtures of complexes *N*,*N*-*cis/trans* [ReOCl­(oz)_2_] (*cis/trans-* **1**). For *trans-* **2**, the catalytic properties in oxyanion (perchlorate and nitrate) reduction were studied. Due to the slow kinetics in the latter, the two cationic complexes [ReO­(**L1**)_2_]­X (X = SO_3_CF_3_®, **3a**; X = O_2_CCF_3_®, **3b**) were synthesized. Cationic triflate complex **3a** showed the highest conversion rates in perchlorate reduction compared to **3b** and chlorido complex *trans-* **2**, corresponding to the weakness of the coordinating anion. A targeted synthesis of the dioxidorhenium­(VI) complex [ReO_2_(**L1**)_2_] (**4**), the product of nitrite (NO_2_®) reduction, allowed for mechanistic and electrochemical investigations. The solid-state structures of complexes *cis-* **2**, *trans-* **2**, **3b**, and **4** were characterized by single-crystal X-ray diffraction.

## Graphical abstract

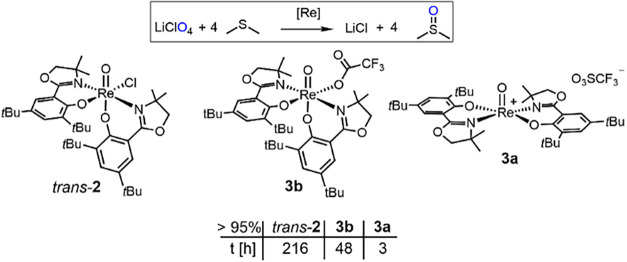

## Introduction

The use as epoxidation catalysts was the initial question that triggered more research into oxidorhenium­(V) complexes by the group of Herrmann in 1996,^1^ with the goal of finding a potential alternative to the highly active catalyst methyltrioxorhenium­(VII) (MTO),^2^ which sometimes caused unwanted ring-opening reactions of epoxides. A second rather unique catalytic reaction enabled by oxidorhenium­(V) complexes was published in 2000 by the group of Abu-Omar. There, the authors could show that the complex [ReOCl­(oz)_2_] (**1**), equipped with the nonmethylated oxazoline-phenol ligand Hoz (Figure 1),^3^ is capable of fully reducing perchlorate via an oxygen atom transfer (OAT) mechanism under mild conditions.^4^ Different sulfides, like SMe_2_, PhSMe, or Ph_2_S, were used as sacrificial oxygen acceptors. In a series of follow-up investigations, a dissociative mechanism, followed by repeated Re­(V) to Re­(VII) redox cycles, was identified, with the first reduction of ClO_4_® to ClO_3_® as the rate-determining step.^5–7^ Very recently, two comprehensive reviews appeared that summarize oxyanion reduction chemistry.^8^

**Figure 1:**
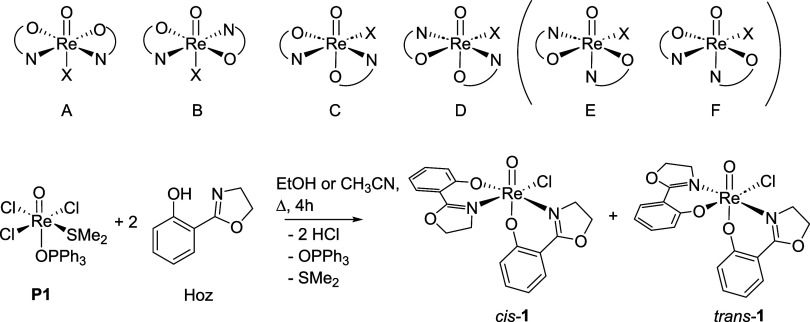
Top: possible stereoisomers for a [ReOX­(ON)_2_] complex. Isomers E and F have not been described yet. Bottom: synthesis of previously published complex [ReOCl­(oz)_2_] (**1**) with the formation of both stereoisomers *N*,*N*-*cis* (*cis-* **1**) and *N*,*N*-*trans* (*trans*-**1**).^4,9^

We also became interested in this unique chemistry of complex **1** and could show that in its synthesis, actually, both *N*,*N*-*cis* (C, Figure 1) and *N*,*N*-*trans* (D, Figure 1) stereoisomers (*cis/trans-* **1**) are formed.^9^ In addition, we showed that *cis-* **1** is inferior in catalytic activity. In addition to the remarkable reactivity toward perchlorate, we also started to investigate the reduction catalysis with nitrate, where the same oxidorhenium­(V) complexes show very promising activity.^10^ Other Fe- and Mo-based nitrate reduction catalysts suffer from their air- and moisture-sensitivity and low catalytic activities.^11^

Based on the importance of stereoisomers in reduction catalysis, a stereoselective synthesis of *N*,*N*-*trans* isomers of such oxidorhenium­(V) complexes garnered interest. The group of Strathmann published two papers on an elegant way to control stereoisomerism in the synthesis of complex **1**.^12,13^ Based on earlier publications by the Abu-Omar group,^5,14^ Strathmann and co-workers could show that the base 2,6-dimethylpyridine (lutidine, lut) is capable of isomerizing the unwanted *N*,*N*-*cis* isomer *cis*-**1** to the desired *N*,*N*-*trans* isomer *trans*-**1** during synthesis.^12^ In contrast to the Hoz ligand, when employing the dimethylated HdmozR ligand class (Figure 2), so far only *N*,*N*-*trans* complexes were obtained, independent of substituents R on the phenol ring or the use of lutidine.^15–17^

**Figure 2:**
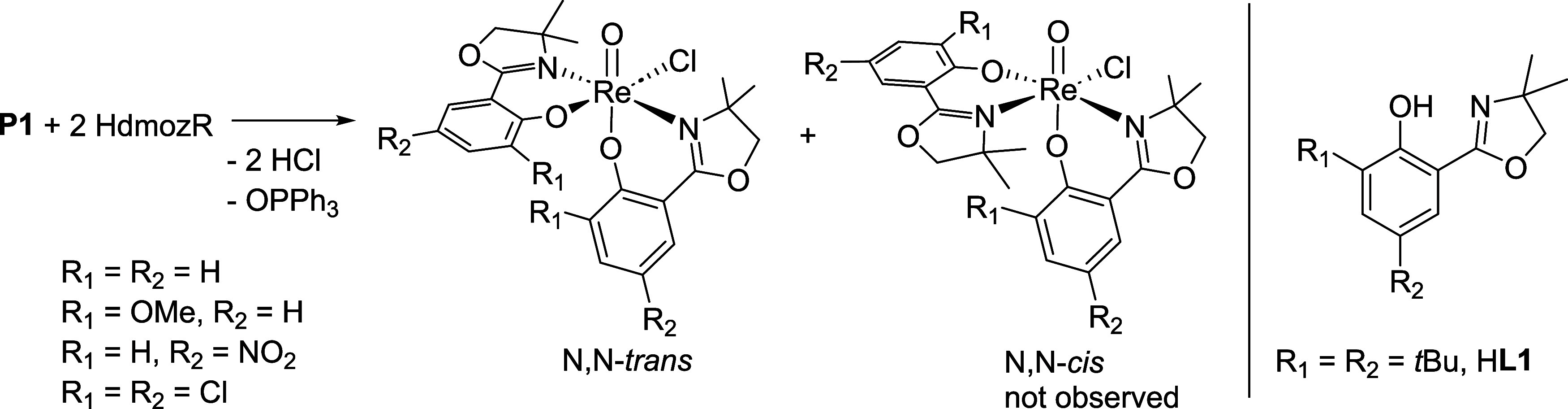
Left: stereoselective synthesis of *N*,*N*-*trans* complexes with previously published HdmozR ligands. Right: substitution pattern of HdmozR ligands, including novel ligand H**L1**.

## Results and Discussion

Synthesis of ligand H**L1** and its pro-ligands H**L1a-b** have already been described in the literature.^18–20^ Our modified procedure (Scheme 1) avoids column chromatography and the use of expensive coupling reagents (e.g., CDI) or toxic solvents (e.g., CCl_4_). Full details are given in the Supporting Information (SI).

**Scheme 1:**
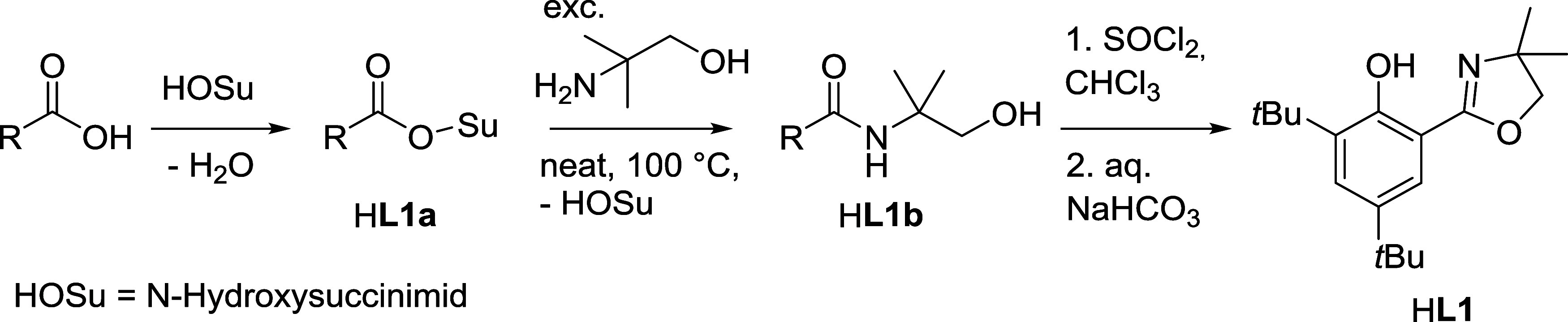
General Scheme for Synthesis of Ligand H**L1**

For H**L1**, we found a literature-known route via a succinimide ester (H**L1a**) to be most convenient.^20^ The benzamide H**L1b** is then obtained by heating in an excess of 2-methylpropane-1-ol (ca. 5 equiv) under neat conditions in almost quantitative yield. Finally, ring closing to furnish the dimethyloxazoline moiety is accomplished by SOCl_2_. Full experimental details of the synthesis and ^1^H NMR spectra of H**L1a**, H**L1b**, and H**L1** are given in the SI (Scheme S1 and Figures S1–S3).

With ligand H**L1** in hand, the synthesis of complex [ReOCl­(**L1**)_2_] (**2**) was undertaken. Initially, precursor [ReOCl_3_(OPPh_3_)­(SMe_2_)] (**P1**) was reacted with two equivalents of H**L1** in the absence of an added base. When this reaction mixture was heated in MeOH to boiling temperatures for 4 h, the solution turned to the expected deep green color. Upon precipitation overnight, a dark green material was isolated that showed good solubility in a variety of standard solvents, including the aliphatic ones (e.g., heptane). The ^1^H NMR spectrum was consistent with a C_1_-symmetric complex, showing two separate sets of ligand signals (Figure S4), suggesting the formation of **2**. Single crystals suitable for X-ray diffraction analysis were obtained from a saturated CH_2_Cl_2_ solution layered with heptane, surprisingly revealing the formation of the *N*,*N*-*cis* isomer (Scheme 3). This represents the first case for an HdmozR ligand, since, as yet, with precursor **P1** and HdmozR ligands (R = H, OMe, NO_2_^15^; di-Cl^17^), only the stereoselective formation of *N*,*N*-*trans* isomers was observed (Figure 1). In our current understanding, the stereochemical outcome is mainly controlled by the *trans* influence of the first coordinated ligand moiety, resulting in intermediate **Int1** (Scheme 2).^15^ In this intermediate, the oxazoline moiety in the equatorial plane can now tighten or loosen the bond to Cl(2), which influences the formation of a *N*,*N*-*cis* or *N*,*N*-*trans* isomer. Because if the phenolate oxygen of the second ligand moiety substitutes either Cl(1) or Cl(3), an *N*,*N*-*trans* isomer is formed, and if Cl(2) is substituted, an *N*,*N*-*cis* isomer is formed.

**Scheme 2:**
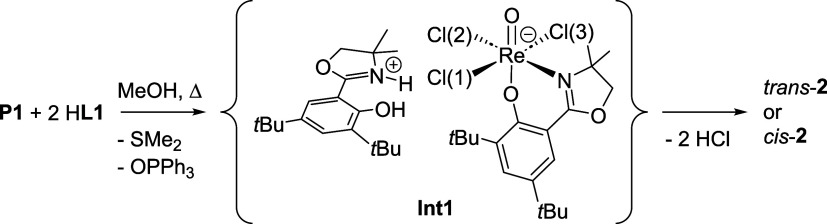
Stereochemical Outcome Controlled by the *trans*Influence Exerted by the **L1** Moiety in **Int1**

Long reaction times of **P1** and H**L1** of 48 h in boiling EtOH finally yielded the desired complex *trans*-**2** in 28% yield (Scheme 3). The electron-donating *t*Bu substituents probably reduce the acidity of the OH proton of H**L1**, which impedes coordination to Re and elimination of HCl. Complex *trans*-**2** showed the same mass spectrum as *cis*-**2**, but a different C_1_-symmetric ^1^H NMR spectrum (Figures S6–S8). Single crystals were obtained at 8 °C from a saturated EtOH solution, which confirmed the *N*,*N*-*trans* isomer (*trans-* **2**, Figure 5). The formation of *trans*-**2** at longer reaction times hinted at thermodynamic-kinetic isomer behavior. Indeed, a mixture of initially 19% *trans-* **2** and 81% *cis-* **2** could be isomerized by dissolving it in toluene-d8 and heating to boiling temperatures. After 16 h of heating, the isomeric ratio increased to 66 and 44%, after 40 h to 78 and 22% and after 64 h, a final mix of 89 to 11% *trans-* **2** to *cis-* **2** was observed by ^1^H NMR spectroscopy. Upon adding either OPPh_3_ or PPh_3_ in excess of 5 equiv to the toluene solution, the observed isomerism was slowed down. These experiments supported that *cis-* **2** is the kinetic product, but *trans-* **2** is the thermodynamic product.

**Scheme 3:**
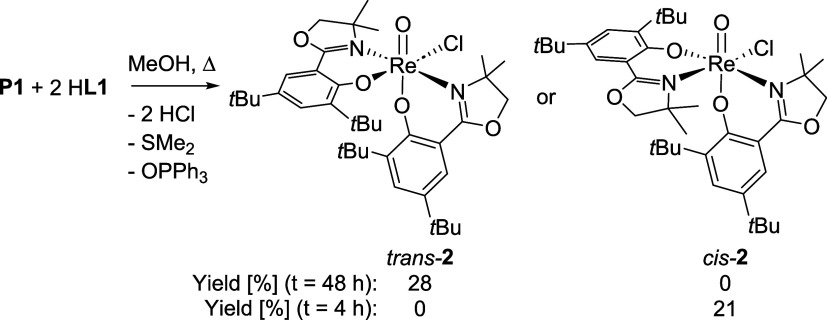
Overview of Reaction Conditions that Lead to Either *trans*-**2** or *cis*-**2** and Respective Yields

Furthermore, this is also supported by DFT calculations (Figures 3 and S25–S31). The data shown in Figure 3 depicts the energies of all six possible isomers of **2** (A–F, Figure 1) as obtained by density functional theory (DFT) using the TMHF^21^ and PBE0-D4 ^22^ functionals and the def2-TZVPP^23^ basis set. Both functionals agree in the energetic order of isomers A–F, with only minor deviations in absolute numbers. DFT therefore confirms the trend we have observed so far for complexes of the HdmozR ligand class, where only *N*,*N*-*trans* complexes (isomer D) were isolated.^9,15,17^ According to the calculations, the *N*,*N*-*cis* isomer C is destabilized by 5.5 to 7.2 kcal/mol when compared to the *N*,*N*-*trans* isomer D. Symmetric isomers A and B are the highest in energy, approximately 24 kcal/mol higher than D. Here, the anionic chlorido ligand would have to coordinate *trans* to the oxido ligand, which is unfavorable. The two O,O-*cis* (E) and O,O-*trans* (F) isomers, which have not been isolated yet in oxidorhenium­(V) complexes, are approximately +15 and +17 kcal/mol higher than D, respectively. Details of DFT calculations and all optimized structures are given in the Experimental Section and SI.

**Figure 3:**
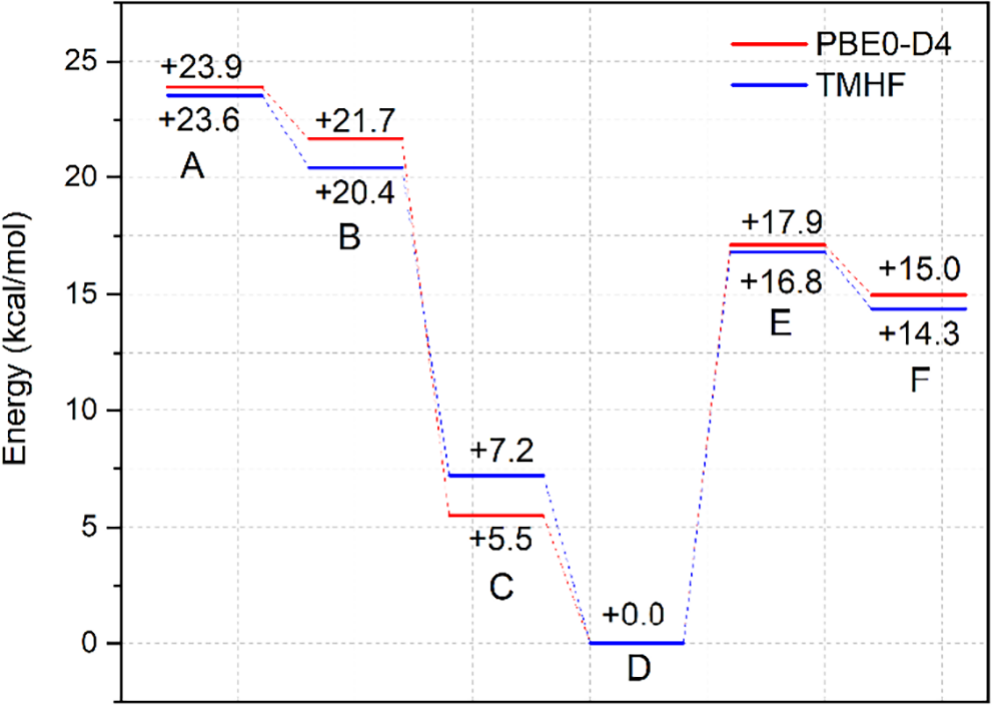
Calculated energies for the six possible isomers A–F of **2**. Energy difference calculated at the TMHF/def2-TZVPP and PBE0-D4/def2-TZVPP levels of theory. All values in kcal/mol.

Perchlorate, as well as catalytic nitrate reduction, follows a dissociative mechanism,^4^ where the chlorido ligand has to decoordinate from the Re center in order to generate a vacant coordination site for the incoming oxyanion substrate. A standard strategy to enhance catalytic activity is to exchange the chlorido ligand in *trans-* **2** with the weakly coordinating anion OTf (= O_3_SCF_3_®), thereby generating a more accessible Re center. Accordingly, *trans-* **2** was reacted with AgOTf to obtain the cationic complex [ReO­(**L1**)_2_]­OTf (**3a**) (Scheme 4). It is interesting to note that boiling in CH_2_Cl_2_ solvent gave the best yields in this reaction, and that at room temperature, no reaction occurred. Reactions in higher-boiling solvents like acetone and acetonitrile gave several unidentified side products. The obtained ^1^H NMR spectrum initially did not show the expected C_2_-symmetric species **3a** (Scheme 4), as in previously synthesized cationic complexes,^10,12,15^ but instead a C_1_-symmetric one (Figure S12), indicating that the equatorial/axial–equatorial arrangement of ligands **L1** of *trans*-**2** had been preserved (**3a**′, Scheme 4). Only after allowing for isomerization overnight at room temperature and dissolving in CH_2_Cl_2_, complex **3a**′ was converted to **3a**, revealing the expected C_2_-symmetric spectrum (Figure S9). In analogy to *cis-* **2** and *trans-* **2**, we also propose **3a**′ to be the kinetic isomer, while **3a** is the thermodynamically favored one. Because of a mix-up of the starting materials, where silver trifluoroacetate (AgTFA) was used instead of silver triflate (AgOTf), complex [ReO­(O_2_CCF_3_)­(**L1**)_2_] (**3b**) (Scheme 4) was cleanly obtained in 91% yield as a green powder. Also, **3b** showed a C_1_-symmetric ^1^H NMR spectrum (Figure S14), but in contrast to **3a**′, no isomerization to a C_2_-symmetric species like **3a** was observed. From the solid-state structure of **3b** (Figure 5), it was revealed that the trifluoroacetate coordinates to the Re center in the cis position to the oxido ligand, thereby preserving the asymmetric coordination of the **L1** moieties, in agreement with the observed NMR spectrum of the solution.

**Scheme 4:**
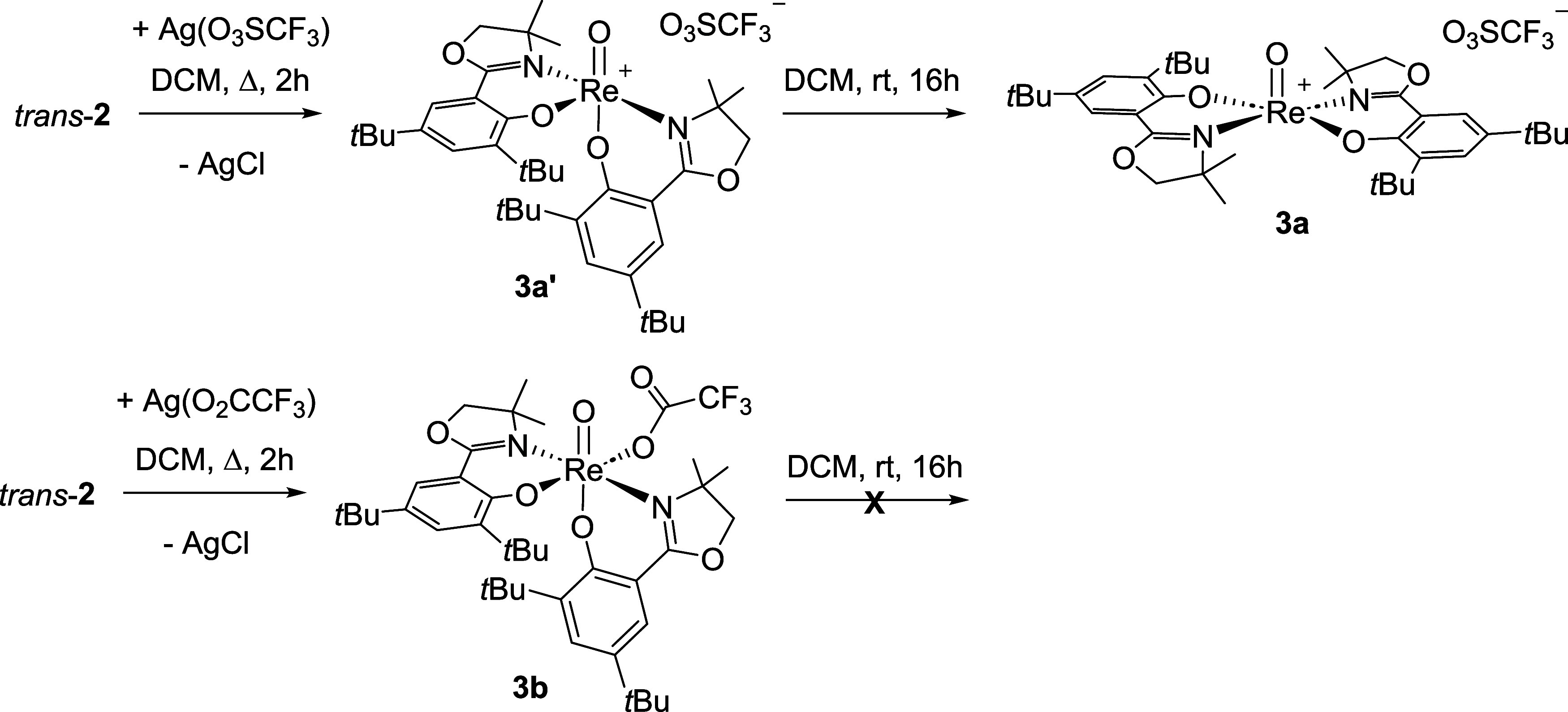
Synthesis of Cationic Triflate Oxidorhenium­(V) Complexes **3a**′ (Kinetic Isomer) and Isomerization to **3a** (Thermodynamic Isomer); Synthesis of Trifluoroacetate Oxidorhenium­(V) Complex**3b**

Several attempts to crystallize **3a**′ or **3a** were undertaken, but no single crystal of high enough quality could be obtained. Instead, only a few crystals of the cationic dioxidorhenium­(VII) complex [ReO_2_(**L1**)_2_]­OTf (**3c**) (Figure S21) could be isolated. The quality of the crystals was not high enough to allow for a full solution of the diffraction data. Nevertheless, the connectivity could be determined, confirming the identity of **3c**. The source of the second oxido ligand is unclear, as no oxidant had been added. However, all crystallization attempts were performed under ambient conditions in the presence of air. Also, the solvents were used directly without predrying or storing under N_2_. In addition to oxygen from air and water from the moist solvents as the potential oxidant, possibly also a disproportionation of Re­(V) complexes **3a/a**′ could lead to **3c**. Despite the unsatisfactory diffraction data, complex **3c** is nevertheless a remarkable species, as in general, only a few structurally characterized examples of such dioxidorhenium­(VII) complexes are known.^24^ In addition, complexes such as **3c** are the proposed intermediates in oxyanion reduction after OAT from the oxyanion to the Re­(V) center has occurred. For all other complexes of the Hoz and Hdmoz ligand family, no such example could be isolated. Only in one case, the Abu-Omar group reported the structure of a similar dioxidorhenium­(VII) complex, where one of the two coordinated oz ligand moieties had decomposed under ring-opening.^7^ Hence, the isolation of **3c** lends more evidence to the generally accepted mechanism of oxyanion reduction under consecutive Re­(V)/Re­(VII) redox cycles.

Furthermore, during perchlorate reduction experiments with *trans-* **2** (see below), single crystals of two decomposition products, namely, [ReO­(**L1**)_2_]­[ReO_4_] (**3d**, Figure 4) and (H_2_ **L1**)­[ReO_4_] (**3e**, Figure 4) were obtained, both containing a ReO_4_® anion. In **3d**, the perrhenate anion is coordinated at the position of the initial chlorido ligand, while, in **3e**, it acts as the counteranion to a protonated (H_2_ **L1**)^+^ ligand. Again, their formation remains unclear; however, the hydrolysis of cationic dioxidorhenium­(VII) complexes like **3c** to give such perrhenate complexes has been previously suggested.^7^ It is interesting to note that complexes **3b**–**d** all remain in the *N*,*N*-*trans* configuration after chloride abstraction, which is important in oxyanion reduction.

**Figure 4:**
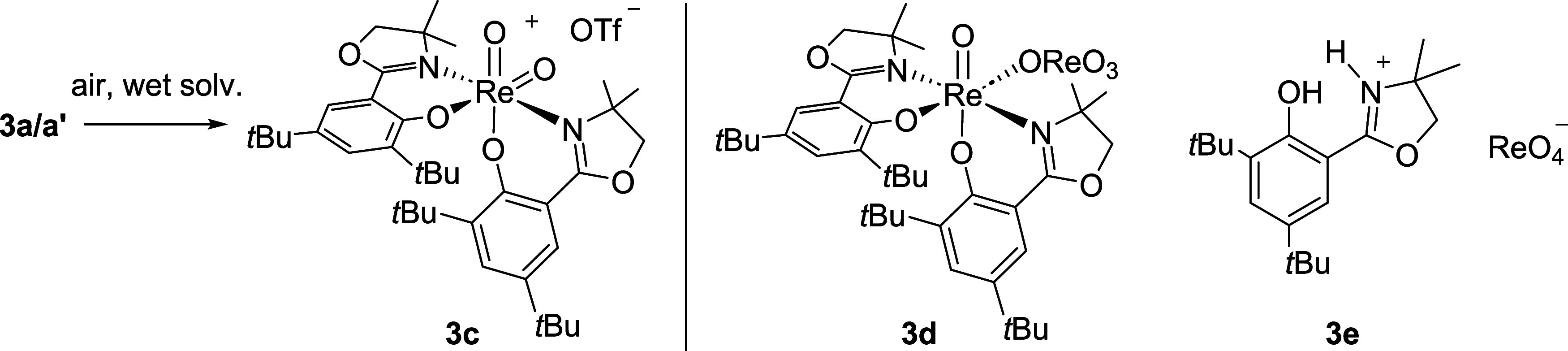
Isolation of complexes **3c**–**3e** from crystallization and perchlorate reduction experiments.

In contrast to the four consecutive 2e̅ reductions cleanly leading from ClO_4_® to Cl̅, in nitrate reduction, the reduction of NO_2_® occurs via a single-electron transfer to give NO. Hence, the resulting, singly oxidized rhenium species would be the neutral, paramagnetic dioxidorhenium­(VI) complex [ReO_2_(**L1**)_2_] (**4**, Scheme 5). Similar paramagnetic rhenium­(VI) complexes have been isolated with the dmoz^10^ and the dmozCl_2_ ^17^ ligand moieties. Indeed, when complex *trans-* **2** is reacted with NO_3_®, a catalytic reduction of NO_3_® to NO_2_® is observed, but over the course of the reaction, the initially green reaction solution turns yellowish, together with the appearance of paramagnetic signals in the ^1^H NMR spectrum. To confirm this and for further mechanistic testing, complex **4** was independently synthesized from a stoichiometric reaction with nitrite (Scheme 5). Yields of **4** were almost quantitative when starting from triflate complex **3a**. While stirring for 2 h, a gradual color change from initially green to orange was observed. The solid-state structure of **4** also confirmed that the *N*,*N*-*trans* configuration was conserved in **4**. As expected for a paramagnetic complex, no meaningful NMR data could be obtained (Figure S17). The symmetric and asymmetric stretching frequencies of the two oxido ligands are observed at 901 and 840 cm^–1^, respectively.

**Scheme 5:**
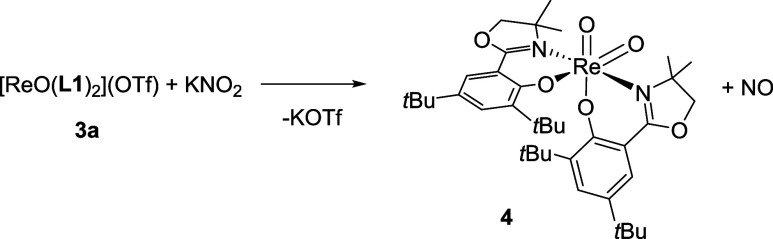
Formation of the Catalytically Inactive Complex **4** during Nitrite Reduction

The formation of such dioxidorhenium­(VI) complexes in the nitrite reduction step is a problem, as the Re center is now in the wrong oxidation state of +VI and cannot perform an OAT to accept SMe_2_ anymore. Thereby, the catalytic cycle stops at this step. In an experiment, isolated complex **4** was mixed with 3 equiv of SMe_2_, and, as also previously observed for complex [ReO_2_(dmoz)_2_],^10^ no formation of OSMe_2_ could be observed.

### Solid-State Structures

Single crystals of *N*,*N*-*cis* [ReOCl­(**L1**)_2_] (*cis*-**2**) were obtained from a CH_2_Cl_2_/heptane mixture of *N*,*N*-*trans* [ReOCl­(**L1**)_2_] (*trans*-**2**) from a saturated EtOH solution, allowing the determination of their structures by X-ray diffraction analysis. Molecular views are given in Figure 5, and selected bond lengths are given in Table 1. Crystallographic data and further bond lengths and angles can be found in the Supporting Information (Tables S3 and S4). In both structures, the Re center is coordinated in a distorted octahedral fashion. Overall, all bond lengths and angles are within the expected ranges (Tables S3 and S4). Whereas the bond lengths to the oxazoline-nitrogen atoms N13 and N33, as well as to the phenolate oxygens O21 and O41 in the two isomers, are similar, there are significant differences in the bond lengths of the oxido and chlorido ligands. In *trans*-**2**, the R1=O1 bond is significantly longer at 1.857(12) Å compared to that in *cis*-**2** at 1.6844(18) Å, which is also closer to other published oxidorhenium­(V) complexes (Table 1). This difference in the Re=O bond distance is remarkable because in both isomers, a phenolate oxygen (O21) is *trans* to the oxido ligand and therefore should induce a similar *trans* influence. When comparing the two Re1–Cl1 bond lengths, *trans*-**2** has a significant shorter distance at 2.325(5) Å compared to *cis*-**2** at 2.3876(7) Å. This is counterintuitive, as in *trans*-**2**, the stronger *trans* influencing phenolate substituent is located *trans* to the chlorido ligand, whereas in *cis*-**2**, only the neutral oxazoline-nitrogen is *trans* to the chlorido ligand. The opposite situation is the case for the two *N*,*N*-*trans*/*cis* isomers of [ReOCl­(oz)_2_] (**1**).^9^

**Figure 5:**
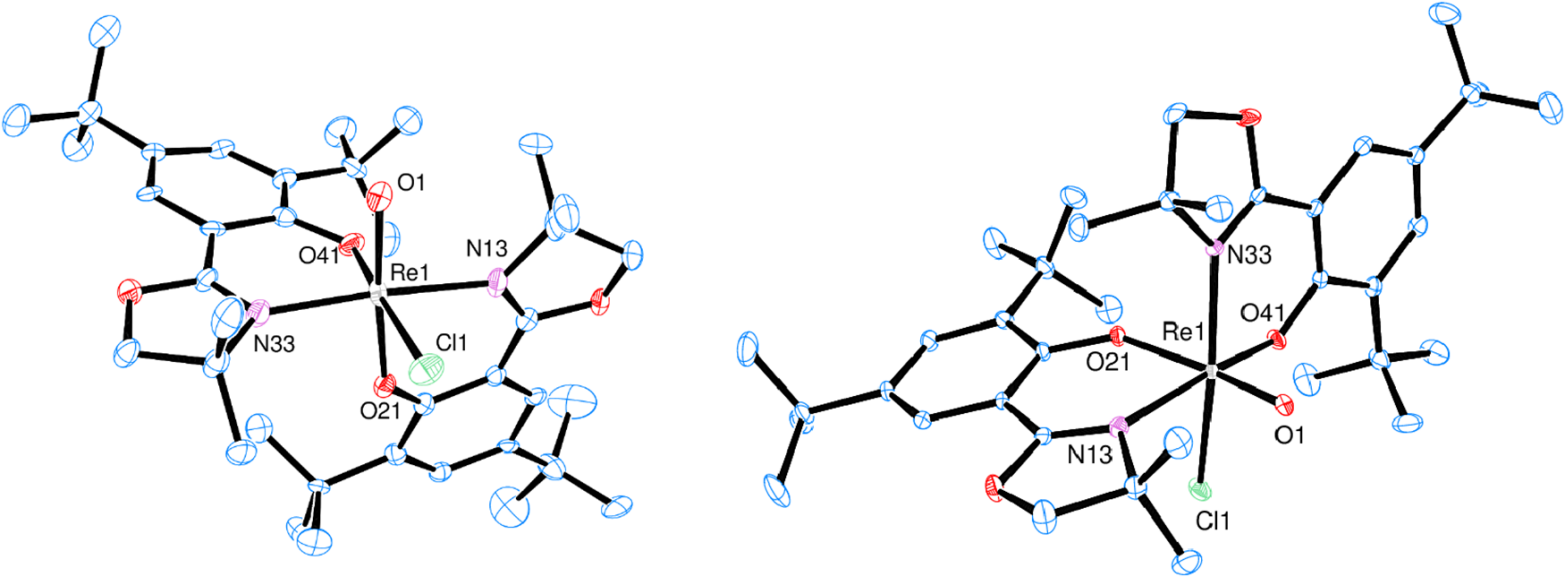
ORTEP plots (50% probability, hydrogen atoms are omitted for clarity). Left, *trans*-**2**. Right, *cis*-**2**. For *trans-* **2**, two independent molecules were found in the unit cell, of which only molecule A is shown.

**Table 1:** Selected Bond Distances of *trans*-**2** and *cis*-**2** and for Comparison of *trans* **-1** and *cis*-1

	Re1=O1	Re1–Cl1	Re1–N13	Re1–N33	Re1–O21	Re1–O41
*trans-* **2** ^a^	1.857(12)	2.325(5)	2.135(12)	2.077(13)	1.967(11)	1.980(11)
*cis-* **2**	1.6844(18)	2.3876(7)	2.197(2)	2.131(2)	1.9630(17)	1.9800(16)
*trans-* **1** ^9^	1.692(3)	2.4093(10)	2.112(2)	2.064(3)	2.001(3)	2.007(3)
*cis-* **1** ^9^	1.689(8)	2.383(3)	2.117(9)	2.116(10)	1.999(7)	1.983(7)

a Of the two independent molecules in the unit cell, only data of molecule A is given (details in the SI).

Single yellow block-shaped crystals of [ReO­(O_2_CCF_3_)­(**L1**)_2_] (**3b**) were obtained from chloroform by slow evaporation, which was suitable for X-ray diffraction analysis. The distorted octahedral coordination around the Re center and the *N*,*N*-*trans* configuration in **3b** are preserved from those of *trans*-**2** (Figure 6, left). The trifluoroacetate ligand coordinates in a η^1^-fashion via O6 of the acetate group to the Re center in the cis position of the oxido ligand with a Re1–O6 distance of 2.088(3) Å (Table 2). The C_1_-symmetric structure observed in the solid state is also observed in solution by NMR spectroscopy. Yellowish-green single crystals of **3d** were grown from a CH_2_Cl_2_/hexane mixture. Structurally, complex **3d** (Figure 6, right) is very similar to **3b**. The perrhenate anion also coordinates in a η^1^-fashion via the O6 of the ReO_4_® group in the cis position of the oxido ligand to the Re center with a Re1–O6 distance of 2.108(2) Å (Table 2). Accordingly, the Re2–O6 bond is slightly elongated at 1.766(2) Å compared to the other three oxido bonds in the perrhenate anion (avg. Re–O distance = 1.701 Å). All of the other bond lengths and angles in **3b** and **3d** are in the expected range (Tables S5 and S6). The occurrence and coordination of perrhenate, most likely due to oxidation and hydrolysis, has been described before.^25,26^ The short Re1–O6 distance points to a strong coordination of the perrhenate anion. In contrast, only three other examples of oxidorhenium­(V) complexes with a trifluoroaceto ligand are characterized, and none is used in catalysis.^26,27^

**Figure 6:**
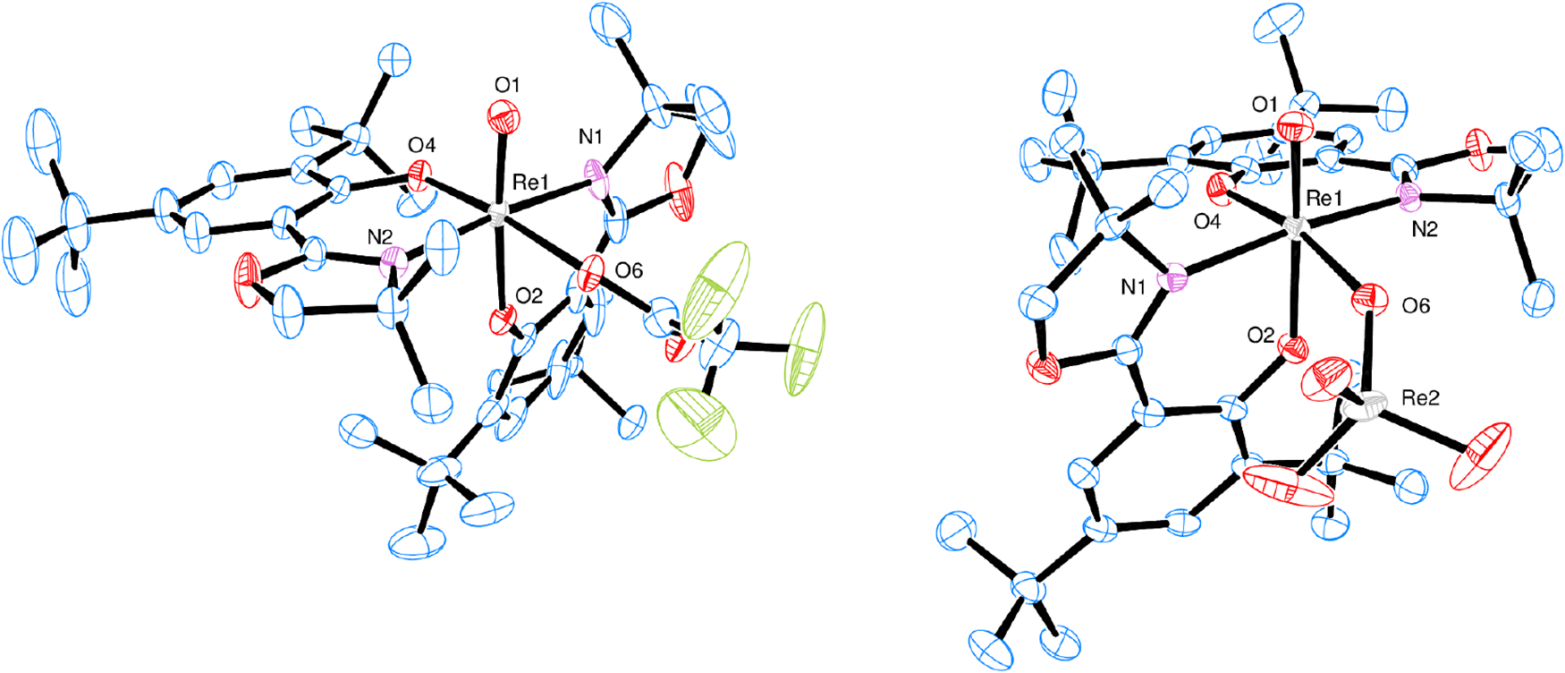
ORTEP plots (50% probability, H atoms are omitted for clarity). Left, **3b.** Disordered atoms of one *t*Bu-group with lower occupancy are not shown. Right, **3d**. Solvent molecule omitted.

**Table 2:** Selected Bond Distances and Angles of Complexes **3b** and **3d**

d (Å)	Re1=O1	Re1–O6	Re1–N1	Re1–N2	Re1–O2	Re1–O4
**3b**	1.677(3)	2.088(3)	2.130(4)	2.083(4)	1.958(3)	2.012(3)
**3d**	1.688(3)	1.766(2)	2.133(3)	2.066(3)	1.970(2)	1.983(2)

**∠ (°)**	O4–Re1–O6	N1–Re1–N2	O1–Re1–O2
**3b**	170.22(13)	172.30(16)	174.90(15)
**3d**	168.24(10)	172.67(11)	172.43(11)

Details on the solid-state structures of **3e** and **4** can be found in the SI, together with information on crystallographic data for all complexes.

### Cyclic Voltammetry

To study the electron-donating influence of ligand **L1** on the rhenium center, complexes *cis-*[ReOCl­(**L1**)_2_] (*cis-* **2**) and *trans-*[ReOCl­(**L1**)_2_] (*trans-* **2**) were investigated by cyclic voltammetry via a standard three-electrode setup in CH_3_CN under inert conditions. Analyte solutions were near 1 mM with (NBu_4_)­PF_6_ used as the supporting electrolyte (0.1 M). The currents *I*_p_ were normalized by the actual concentrations to allow for better comparability. Half-wave potentials *E*_1/2_ (*E*_1/2_ = (*E*_p,c_ + *E*_p,a_)/2) are given in Table 3.

**Table 3:** Comparison of Redox Potentials E_1/2_ [V] at 200 mV s^–1^ with Previously Published Oxidorhenium­(V) Complexes

*E*_1/2_ [V]	*N*,*N*-*trans*	*N*,*N*-*cis*	refs
[ReOCl(dmoz*t*Bu_2_)_2_] (**2**)	0.48	0.46	^a^
[ReOCl(oz)_2_] (**1**)	0.58	0.58	^15^
[ReOCl(dmoz)_2_]	0.64		^15^
[ReOCl(dmozOMe)_2_]	0.61		^15^
[ReOCl(dmozNO_2_)_2_]	0.92		^15^
[ReOCl(dmozCl_2_)_2_]	0.85		^17^

a This work.

The cyclovoltammograms given in Figure 7 reveal that the *N*,*N*-*cis*/*trans* isomerism of **2** has no measurable impact on the redox potential of the rhenium center. The same observation was made for the isomers of [ReOCl­(oz)_2_] (**1**).^15^ An overview of redox potentials is given in Table 3, showing that the electronic influence of the ligands is reflected well in the electrochemistry of the respective complexes. Indeed, complex **2**, with the electron-donating ligand **L1**, shows the lowest redox potential at an average of 0.48 V. The parent complex [ReOCl­(dmoz)_2_] without substituents on the phenol ring shows a reversible half potential of *E*_1/2_ = 0.64 V.^15^ The cyclovoltammograms of both **3a** and **4** depicted irreversible behavior.

**Figure 7:**
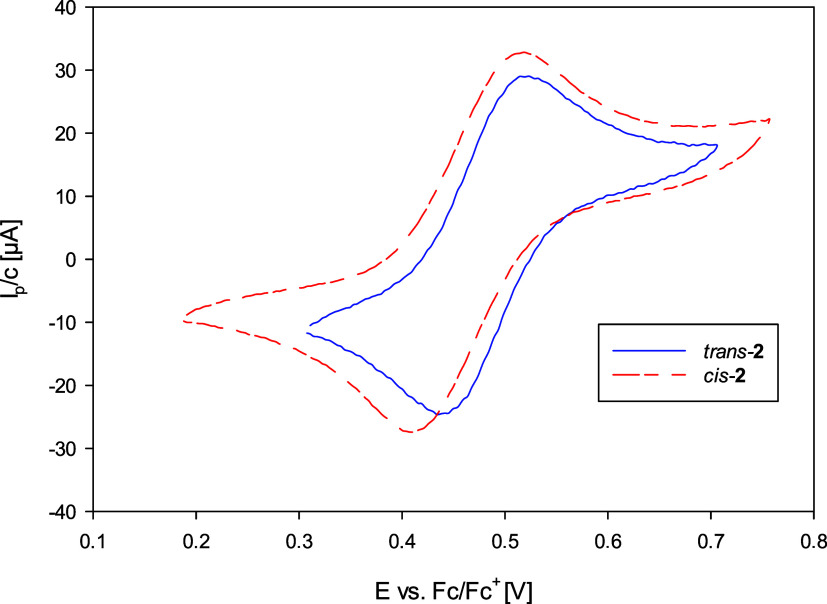
Comparison of cyclovoltammograms of *cis*-**2** and *trans-* **2**.

### Oxyanion Reduction

Experiments for the catalytic reduction of perchlorate were conducted under standard conditions (25 °C, CD_3_CN/D_2_O = 95/5 vol %), with either 10 or 3.2 mol % of catalyst and 4 equiv of SMe_2_. The progress of the catalytic reaction was followed by the conversion of SMe_2_ to OSMe_2_ via ^1^H NMR spectroscopy (Scheme 6).

**Scheme 6:**
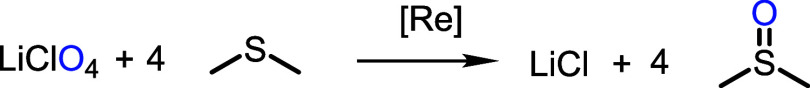
Catalytic Perchlorate Reduction Using SMe_2_ as a Sacrificial Oxygen Acceptor

In perchlorate reduction, four consecutive oxygen atom transfer (OAT) steps occur to fully reduce ClO_4_® to Cl̅. During catalysis, the rhenium center cycles between Re­(V) and Re­(VII).^4^ Under the conditions mentioned above, the *N*,*N*-*trans* isomer *trans*-**2** showed catalytic activity, although with very slow kinetics (Table 4), but exceptional stability under reaction conditions (Figure 8). In the first 24 h, a conversion of only 37% to DMSO was observed. In comparison, complex *trans-* **1** reaches full conversion after 4 h at only 3.2 mol % catalyst loading (Table 4).^4^ However, we kept monitoring the reaction, and after 216 h or 9 days, the reaction had reached full conversion of >95%. The rate-determining step was previously identified as being the first OAT from ClO_4_® to the Re­(V) center.^4^ In complex *trans-* **2**, this step might be associated with a higher activation barrier, as the Re center is more electron-rich compared to [ReOCl­(dmoz)_2_] or *trans-* **1**. Such stronger binding would be detrimental to the dissociative mechanism of perchlorate reduction. A comparison of the Re1–Cl1 bond lengths shows that in *trans-* **2**, the bond is indeed shorter at 2.325(5) Å compared to those in *trans-* **1** (2.4093(10) Å^9^) and [ReOCl­(dmoz)_2_] (2.440(2) Å^15^). In addition, the isolation of **3c** in the fully oxidized Re­(VII) state also hints at the stabilization of the high oxidation state, which would again slow down OAT to SMe_2_.

**Figure 8:**
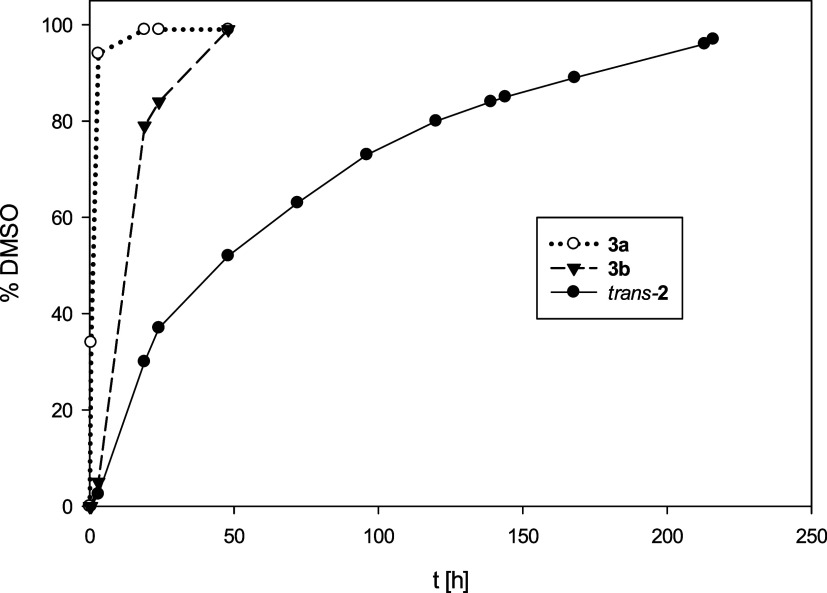
Catalytic perchlorate reduction with *trans-* **2**, **3a**, and **3b** (10 mol %, 4 equiv. SMe_2_, rt, CD_3_CN/D_2_O = 95/5).

**Table 4:** Comparison of Catalytic Perchlorate Reduction between *trans-*2, **3a**, and **3b** (4 Equiv. SMe_2_, rt, CD_3_CN/D_2_O = 95/5) and Previously Published Oxidorhenium­(V) Complexes

	mol %	*t* [h]	[%]	refs
*trans*-**2**	10	24	37^a^	^c^
**3a**	10	3	>95	^c^
**3b**	10	24	84^b^	^c^
**3a**	3.2	24	62	^c^
**3b**	3.2	24	24	^c^
*trans-* **1**	3.2	4	>99	^4^
*cis-* **1**	3.2	24	33	^15^
[ReOCl(dmoz)_2_]	3.2	24	75	^15^
[ReOCl(dmozOMe)_2_]	3.2	24	78	^15^
[ReOCl(dmozNO_2_)_2_]	3.2	24	48	^15^
[ReOCl(dmozCl_2_)_2_]	3.2	24	12	^17^

a 216 h, >95%.

b 48 h, >95%.

c This publication.

In order to accelerate the catalytic reaction, the tightly bound chlorido ligand in *trans*-**2** was replaced with the weakly coordinating triflate anion in complex [ReO­(**L1**)_2_]­(OTf) (**3a**). Indeed, we were delighted to observe that triflate complex **3a** now showed significantly increased catalytic activity compared to *trans*-**2** (Figure 8). After 3 h, already 94% of SMe_2_ was converted to OSMe_2_, a full 8 days faster than the chlorido complex *trans*-**2**. This rate of conversion was independent of the isomer used, namely, kinetic isomer **3a**′ or **3a**. To further verify the importance of the availability of a vacant coordination site, an accidentally synthesized trifluoroacetate complex [ReO­(O_2_CCF_3_)­(**L1**)_2_] (**3b**) was tested. The trifluoroacetate anion (TFA) does not behave as a truly “weakly coordinating anion”, as observed by the C_1_-symmetric ^1^H NMR spectrum (Figure S15), as well as in the solid-state structure (Figure 6), which shows a bond between Re1 and O6. Accordingly, catalytic activity between those of triflate complex **3a** and chlorido complex *trans*-**2** was observed, taking 48 h to reach >95% conversion, reflecting a weaker bonding as compared to the chlorido ligand in *trans*-**2**.

There are various reviews available on the subject of weakly coordinating anions (WCAs),^28^ and also systematic studies of the effect of various WCAs in catalysis have been published.^29^ However, much fewer investigations are available on the direct comparison of OTf vs TFA complexes in the same catalytic reaction.^30^ In the case of homogeneous gold catalysts [L-Au]­X (with X = OTf or TFA), a clear trend for the influence of the anion X̅ could not be established, as there was also a dependence on the specific substrate or other ligands present. In the following example, there was no catalytic difference at all. The complexes [Fe­(OTf)_3_] and [Fe­(TFA)_3_] were used in the Hantzsch and Biginelli reactions, but showed the same reactivity.^31^ In contrast, the data from our investigation allow for determining a clear relationship between the strength of coordination of the anion to the catalytic activity of the complex in perchlorate reduction. The stronger the anion is coordinated, the slower the catalysis in the order of Cl̅ (*trans-* **2**) < O_2_CCF_3_® (**3b**) < O_3_SCF_3_® (**3a**).

In a second round of experiments, the influence of the added water content on the perchlorate reduction activity was tested. The experiments with catalyst *trans-* **2** (10 mol %) were performed using 0, 10, and 20 vol% of added D_2_O to the solvent CD_3_CN. An initial trend could be observed, confirming the importance of added water to the reactivity (Figure 9). Best results were obtained with the addition of 5 and 10 vol% D_2_O. In the absence of added D_2_O, the reaction is significantly slower, only reaching <5% conversion. Water is important for stabilizing all of the cationic intermediates and transition states that form during the catalytic cycle, as previous DFT calculations for [ReOCl­(dmoz)_2_] had shown.^10^ Interestingly, at 20 vol% D_2_O, the conversion decreased again. Here, potentially solvation effects become a problem by inhibiting access to the vacant coordination site on the Re ion.

**Figure 9:**
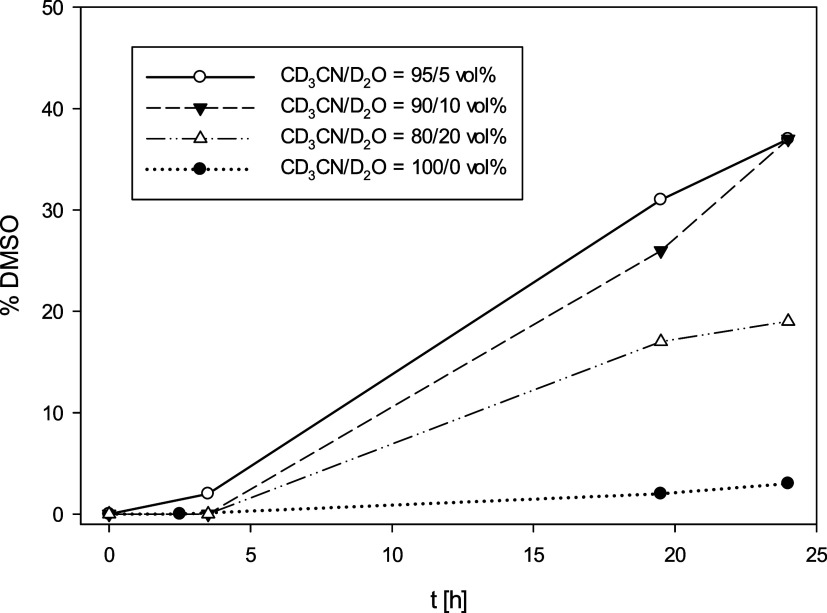
Dependence of perchlorate reduction activity on water content (*trans*-**2**, 10 mol %, 4 equiv. DMS, 25 °C).

## Conclusion

Oxidorhenium­(V) complexes containing phenol–dimethyloxazoline ligands are capable of both reducing harmful and kinetically very stable anions such as nitrate and perchlorate via OAT reactions under mild and ambient conditions, as well as catalytically epoxidize cyclooctene. Here, complex [ReOCl­(**L1**)_2_] (**2**) is equipped with two *t*Bu bearing ligands, acting as electron-donating groups and increasing the electron density on the Re center. This led to the first observation of the catalytically important Re­(VII) cation [ReO_2_(**L1**)_2_]­(OTf) (**3c**). In contrast to previously published complexes of the type [ReOCl­(dmozR)_2_], both *N*,*N*-*trans* (*trans*-**2**) and *N*,*N*-*cis* (*cis*-**2**) isomers could be isolated, with the former corresponding to the thermodynamic and the latter to the kinetic product, as supported by both DFT calculations and isomerization experiments. The complex *trans*-**2** was tested for the reduction of perchlorate, where it should have slow kinetics but long stability. A full 9 days were needed for complete conversion. As perchlorate reduction operates under a dissociative mechanism, the cationic triflate complex **3a** and trifluoroacetate complex **3b** were synthesized, which led to an extreme acceleration of catalysis. A strong correlation between weakness of the coordinating anion and activity in perchlorate reduction was observed: Cl̅ (*trans-* **2**) < O_2_CCF_3_® (**3b**) < O_3_SCF_3_® (**3a**).

## Experimental Section

### General

Ligand H**L1** has been previously published.^17^ The rhenium precursor [ReOCl_3_(OPPh_3_)­(SMe_2_)] ^14,32^ **P1** and ligand H**1b** ^18,20^ have been synthesized according to literature procedures with some modifications (see SI). Chemicals were purchased from commercial sources and were used without further purification. NMR spectra were recorded with a Bruker (300 MHz) instrument. Chemical shifts are given in parts per million and are referenced to residual protons in the solvent. Signals are described as s (singlet), bs (broad singlet), d (doublet), dd (doublet of doublet), t (triplet), qd (quaternary doublet), and m (multiplet), and coupling constants (*J*) are given in Hertz (Hz). Mass spectra were recorded with an Agilent 5973 MSD (Direct Probe) using the EI ionization technique. Samples for infrared spectroscopy were measured on a Bruker Optics α FT-IR Spectrometer equipped with an ATR diamond probe head. GC-MS measurements were performed on an Agilent 7890 A with an Agilent 19091J–433 column coupled to a mass spectrometer type Agilent 5975 C. Elemental analyses were carried out using a Heraeus Vario Elementar automatic analyzer at the University of Technology Graz. No uncommon hazards are noted.

#### Synthesis of *N*,*N*-*cis* [ReOCl­(**L1**)_2_] (*cis* **-2**)

Ligand H**L1** (982 mg, 3.24 mmol, 2.1 equiv) and precursor [ReOCl_3_(OPPh_3_)­(SMe_2_) (**P1**)] (1 g, 1.54 mmol, 1 equiv) were refluxed in 30 mL MeOH for 4 h. After concentration to approximately 5 mL, the reaction solution was topped with 5 mL of Et_2_O. Cooling to 8 °C for 24 h led to the precipitation of crude *cis-* **2** as a green crystalline solid. Washing with small amounts of cold Et_2_O led to analytically pure *cis-* **2**. Yield: 272 mg (0.32 mmol, 21%). ^1^H NMR (300 MHz, Chloroform-d) δ 7.72 (d, *J* = 2.5 Hz, 1H), 7.61 (d, *J* = 2.6 Hz, 1H), 7.55 (d, *J* = 2.5 Hz, 1H), 7.28 (d, *J* = 2.5 Hz, 1H), 4.54 (d, *J* = 8.2 Hz, 1H), 4.40 (d, *J* = 8.2 Hz, 1H), 4.39 (d, *J* = 8.3 Hz, 1H), 3.97 (d, *J* = 8.3 Hz, 1H), 1.89 (s, 3H), 1.87 (s, 3H), 1.58 (s, 9H), 1.47 (s, 3H), 1.33 (s, 9H), 1.27 (s, 9H), 1.12 (s, 3H), 1.05 (s, 9H). ^13^C NMR (75 MHz, Chloroform-d) δ 172.71, 170.99, 167.54, 160.44, 141.10, 140.61, 139.39, 138.46, 131.40, 131.06, 125.33, 123.27, 109.98, 108.92, 79.15, 78.40, 78.24, 74.54, 35.88, 35.07, 34.65, 31.61, 31.48, 30.34, 29.72, 27.11, 26.53, 25.82; ATR-IR (cm^–1^): 2957.5 (m), 1606.4 (m) and 1546.7 (m) (ν C=N), 1250.6 (s), 1112.9 (m), 965.1 (vs) (ν Re=O), 849.6 (s), 735.1 (s), 540.6 (m); EI-MS (*m*/*z*): 842.7 [M^+^]. UV–vis (CH_2_Cl_2_) λ_max_, nm (ε): 590 (135). Anal. Calcd. for C_38_H_56_ClN_2_O_5_Re (842.5 g/mol): C 54.17, H 6.70, N 3.32; found: C 53.52, H 5.96, N 3.11.

#### Synthesis of [ReOCl­(**L1**)_2_] (*trans* **-2**)

H**L1** (1.0 g, 3.29 mmol, 2 equiv) and precursor [ReOCl_3_(OPPh_3_)­(SMe_2_)] (**P1**) (1.07 g, 1.64 mmol, 1 equiv) were heated to reflux temperatures in 30 mL of ethanol for 48 h, resulting in a light green precipitate. Additional product could be obtained upon concentration of the reaction solution and precipitation at 8 °C. Yield: 387 mg (0.46 mmol, 28%). ^1^H NMR (300 MHz, Chloroform-d) δ 7.68 (d, *J* = 2.6 Hz, 1H), 7.59 (d, *J* = 2.5 Hz, 1H), 7.29 (d, *J* = 2.6 Hz, 1H), 7.19 (d, *J* = 2.5 Hz, 1H), 4.65 (d, *J* = 8.1 Hz, 1H), 4.56 (d, *J* = 8.1 Hz, 1H), 4.50 (d, *J* = 8.1 Hz, 1H), 4.33 (d, *J* = 8.2 Hz, 1H), 2.00 (s, 3H), 1.89 (s, 3H), 1.84 (s, 3H), 1.74 (s, 3H), 1.25 (s, 9H), 1.24 (s, 9H), 1.08 (s, 9H), 0.96 (s, 9H). ^13^C NMR (75 MHz, Chloroform-d) δ: 137.20, 130.62, 130.34, 125.43, 123.86, 31.55, 31.41, 29.71, 29.48, some C are obscured. ATR-IR (cm^–1^): 2950.1 (m), 2903.3 (w), 2867.8 (w), 1613.8 (m) and 1570.3 (m) and 1537.9 (m) (ν C=N), 1443.6 (m), 1432.6 (m), 1246.0 (m), 1193.4 (s), 957.9 (s) (ν Re=O), 840.3 (s), 730.4 (m), 534.4 (m), 448.5 (m); EI-MS (*m*/*z*): 842.7 [M^+^]; UV–vis λ_max_ (ε) (nm, l/mol cm, CH_2_Cl_2_): 664 (60); Anal. Calcd. for C_38_H_56_ClN_2_O_5_Re (842.5 g/mol): C 54.17, H 6.70, N 3.32; found: C 54.02, H 6.75, N 3.29.

#### Synthesis of [ReO­(**L1**)_2_]­(O_3_SCF_3_) (**3a**)

[ReOCl­(**L1**)_2_] (*trans* **-2**) (500 mg, 0.59 mmol, 1.0 equiv) and silver trifluoromethanesulfonate (AgOTf) (180 mg, 0.70 mmol, 1.2 equiv) were refluxed in 30 mL of DCM for 2 h. Precipitated AgCl was removed by filtration. After the reaction solution had been left to stand for 24 h, all of the kinetic product **3a’** was isomerized to the thermodynamic product **3a**, which was also observed as the deepening of the green color (**3a**). After the removal of the solvent and washing with Et_2_O, **3a** was obtained in analytically pure form as a deep green crystalline solid. Yield: 508 mg (0.53 mmol, 90%). ^1^H NMR (300 MHz, Chloroform-d) δ 7.79 (d, *J* = 2.3 Hz, 2H), 7.71 (d, *J* = 2.3 Hz, 2H), 4.79 (d, *J* = 9.2 Hz, 2H), 4.64 (d, *J* = 9.2 Hz, 2H), 1.83 (s, 6H), 1.77 (s, 6H), 1.32 (s, 18H), 1.03 (s, 18H). ^13^C NMR (75 MHz, CDCl_3_) δ 171.42, 160.02, 147.02, 139.35, 133.90, 123.92, 113.23, 82.33, 74.20, 34.91, 34.76, 31.33, 29.12, 25.51, some C are obscured; ATR-IR (cm^–1^): 2657.3 (m), 1517.2 (m) (ν C=N), 1388.1 (bs), 1196.1 (m), 1116.5 (s), 1028.3 (m), 959.9 (s) (ν Re=O), 844.3 (s), 634.8 (s), 550.0 (m); EI-MS (*m*/*z*): 956.6 [M^+^]. UV–vis λ_max_ (ε) (nm, l/mol cm, CH_2_Cl_2_): 550 (140). Anal. Calcd. for C_39_H_56_ F_3_N_2_O_8_ReS^+^ (956.1 g/mol): C 48.99, H 5.90, N 2.93, S 3.35; found: C 47.00, H 5.60, N 2.68, S 3.19.

#### Synthesis of [ReO­(O_2_CCF_3_)­(**L1**)_2_] (**3b**)

A 33.1 mg (150 mmol, 1.1 equiv) of silver trifluoroacetate (AgTFA) and 115 mg of *trans-* **2** (136 mmol, 1 equiv) were dispersed in 10 mL of CH_2_Cl_2_ and stirred at reflux temperature for 4 h. The precipitated AgCl was removed by filtration, and the remaining reaction solution evaporated to dryness to yield 114 mg of **3b** (124 mmol, 91% yield). ^1^H NMR (300 MHz, CDCl_3_) δ: 7.70 (d, *J* = 2.6 Hz, 1H), 7.60 (d, *J* = 2.6 Hz, 1H), 7.33 (d, *J* = 2.6 Hz, 1H), 7.20 (d, *J* = 2.5 Hz, 1H), 4.66 (d, *J* = 8.4 Hz, 1H), 4.58 (d, *J* = 8.1 Hz, 1H), 4.50 (d, *J* = 8.1 Hz, 1H), 4.42 (d, *J* = 8.4 Hz, 1H), 1.82 (s, 3H), 1.74 (s, 3H), 1.72 (s, 3H), 1.63 (s, 3H), 1.24 (d, *J* = 1.5 Hz, 17H), 1.01 (d, *J* = 3.7 Hz, 17H). ^13^C NMR (75 MHz, Chloroform-*d*) δ 175.30, 173.23, 166.61, 166.06 (q, ^2^ *J*(^19^F,^13^C) = 37.01 Hz, -O_2_ *C*CF_3_), 160.37, 140.89, 139.98, 139.46, 136.83, 131.04, 130.24, 125.44, 124.24, 114.09 (q, ^1^ *J*(^19^F,^13^C) = 291.05 Hz, -O_2_C*C*F_3_), 110.61, 110.30, 81.52, 79.00, 72.88, 35.35, 35.00, 34.58, 34.22, 31.47, 29.77, 29.38, 27.69, 27.42, 26.61; ^19^F NMR (282 MHz, CDCl_3_) δ −75.38 (s). EI-MS (*m*/*z*): 920.7 [M^+^]. ATR-IR (cm^–1^): 2959.3 (m), 1719.1 (m), 1575.7 (m) (ν C=N), 1381.1 (m), 1183.0 (s, ν O_2_CCF_3_), 1141.7 (m), 1116.0 (m), 958.3 (m, ν Re=O), 722.9 (m), 538.6 (m). Anal. Calcd. for C_40_H_56_F_3_N_2_O_7_Re (920.1 g/mol): C 53.14, H 6.24, N 3.10; found: C 52.22, H 6.18, N 3.16.

#### Synthesis of [ReO_2_(**L1**)_2_] (**4**)

[ReO­(**L1**)_2_]­(OTf) (**3a**) (87.5 mg, 0.092 mmol, 1 equiv) and potassium nitrite (exc.) were stirred in 4 mL of ACN with 5 vol% H_2_O for 2 h. A change in color from green to orange was observed. The solvents were removed completely and the crude product was washed with small amounts of cold water and Et_2_O, giving **4** as an orange precipitate. Yield: 69 mg of **4** (0.084 mmol, 91%). ATR-IR (cm^–1^): 2951.9 (m), 1612.7 (s), 1568.0 (m) (ν C=N), 1251.3 (bs), 1193.3 (m), 901.7 and 839.8 (ν Re=O), 797.0 (m), 525.7 (m) EI-MS (*m*/*z*): 823.5 [M^+^]. UV–vis (CH_3_Cl) λ_max_, nm (ε): 495 (872). Anal. Calcd. for C_38_H_56_N_2_O_6_Re (823.1 g/mol): C 55.45, H 6.86, N 3.40; found: C 54.51, H 6.53, N 3.30.

### Computational Details

All geometries were optimized by using the r^2^SCAN functional in combination with the D4 dispersion correction. Frequency calculations were performed to ensure that the optimization has converged to a stationary point. Energy differences for the isomers 2A–F were calculated at the optimized geometries using the PBE0 functional in combination with the D4 dispersion correction and the TMHF functional. Theoretical absorption spectra were calculated using the correlation-kernel augmented eigenvalue self-consistent GW-Bethe-Salpeter-equation method (evGW-cBSE). As a reference Kohn–Sham state for evGW-cBSE, the TMHF functional was used due to its robust performance. The def2-TZVPP basis set was used in all calculations. For Re, an effective core potential (ECP) describing 60 core electrons was used. DFT calculations were converged to changes of 10^–8^ a.u. in the energy and 10^–7^ a.u. in the density matrix. The evGW quasiparticle energies were converged to 10^–5^ a.u. In the evGW calculations, 7 states around the Fermi level were optimized, and the remaining quasiparticle energies were shifted accordingly (“scissoring”). All calculations were performed using a development version of Turbomole V7.7.

## Supplementary Material



## Notes

Details of the synthesis of ligand H**L1** via intermediate products H**L1a** and H**L1b**; depiction of ^1^H NMR spectra of H**L1a**, H**L1b**, H**L1**, and **4**; ^1^H and ^13^C NMR spectra of *cis-* **2** and *trans-* **2**; ^1^H, ^13^C, and ^19^F NMR spectra of **3a**, **3a’**, and **3b**; metadata is deposited at Zenodo under the DOI 10.5281/zenodo.15680558; details on DFT computations and calculated structures of the six possible isomers A–F are given; details on crystallographic data acquisition and refinement as well as selected bond lengths and angles of *cis*-**2**, *trans*-**2**, **3b**, **3d**, and **4** are given (PDF)
