# Supplementary material for: Electron-Donating Ligand in Oxidorhenium(V) Chemistry: Consequences for Isomerism and Catalyst Properties
Source: Inorg Chem. 2026 Jan 20;65(4):2295–306. doi: 10.1021/acs.inorgchem.5c04871 (PMC12869481; doi:10.1021/acs.inorgchem.5c04871)
Supplement: Supplementary file 1 [file ic5c04871_si_001.pdf]

Supporting Information for

An electron-donating ligand in oxidorhenium(V) chemistry: consequences for isomerism and catalyst properties

Cornelia Rom,† Christof Holzer,‡ Antoine Dupé,† Ferdinand Belaj,† Nadia C. Mösch-Zanetti†,* and Jörg A. Schachner,†,*

†Institute of Chemistry, University of Graz, Schubertstr. 1, 8010 Graz, Austria.

‡Institute of Quantum Materials and Technologies, Karlsruhe Institute of Technology, Wolfgang-Gaede-Str. 1, 76131 Karlsruhe, Germany.

corresponding author: joerg.schachner@uni-graz.at

Improved synthesis of HL1 (Scheme S1). All steps can be performed in laboratory grade solvents without the need for inert atmosphere or column chromatography. An alternative synthesis requiring exclusion of air and moisture using CDI in the first step instead of *N*-hydroxysuccinimide (HOSu) and column chromatography of ligand HL1 had been previously published.[1]

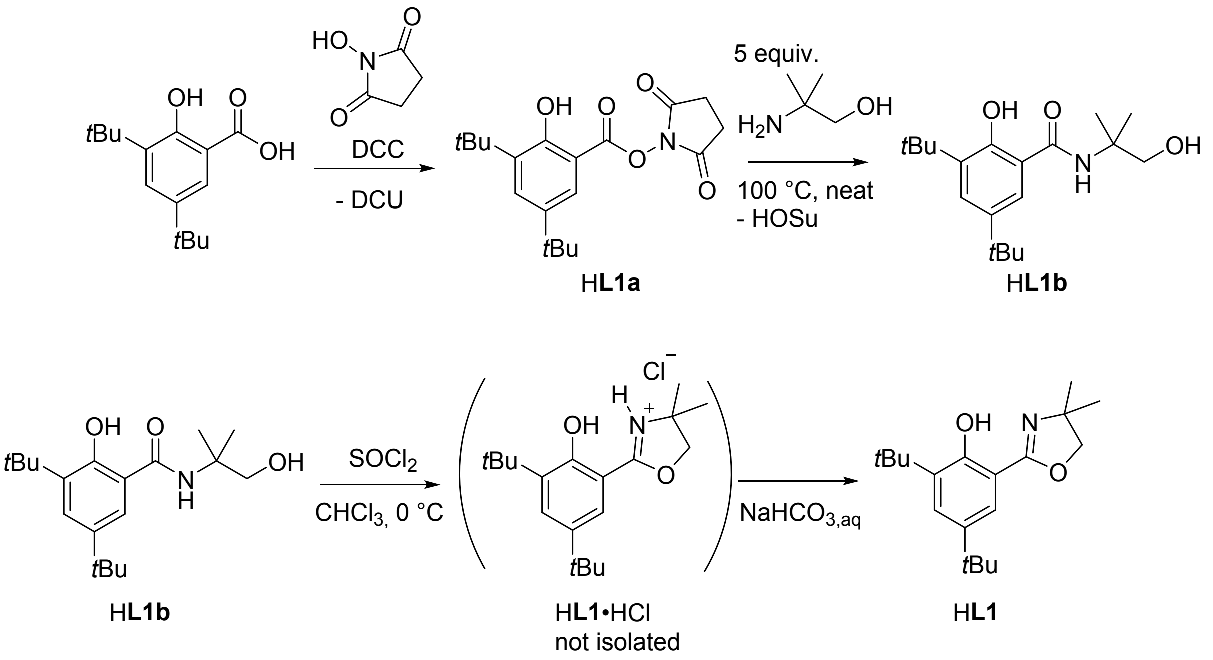

Scheme S1. Full synthesis of HL1.

The synthesis of succinimide ester **HL1a** has been previously published.[2]

Synthesis of **HL1b**. 5.26 g of **HL1b** (15.14 mmol, 1 equiv.) and 2-methyl-2-amino-propanol (6.75 g, 75.70 mmol, 5 equiv.) were mixed and heated to 100 °C in an open flask (no reflux condenser) for 6 h. Reaction progress can be monitored by TLC (mini-work up with 2M HCl/Et_2O). The resulting oil was purified by acidic work-up with heptane and 2M HCl; the generated white precipitate of **HL1b** was filtered with a Buchner funnel and dried. R_f (product, Cy/EE 1/1) = 0.5; Yield = 3.5 g (11.01 mmol, 72.67%). ^{1}H NMR (300 MHz, Chloroform-d) δ 12.29 (br. s, 1H), 7.46 (d, *J* = 2.3 Hz, 1H), 7.07 (d, *J* = 2.3 Hz, 1H), 6.30 (s, 1H), 3.72 (s, 2H), 1.44 (s, 6H), 1.43 (d, *J* = 0.7 Hz, 1H), 1.42 (s, 9H), 1.31 (s, 9H). ATR-IR (cm^{-1}): 3280 (m), 2954 (s), 1580 (s) and 1548 (s) C=N, 1434 (s), 1337 (s), 1058 (s), 799 (vs), 727 (m), 683 (m), 639 (m), 480 (m); EI-MS (*m*/*z*): not applicable, as only the product mass of 303 of **HL1** is visible due to thermal ring closing.

Synthesis of **HL1**. 310 mg of **HL1b** (0.96 mmol, 1 equiv.) were dissolved in 4 ml $CHCl_3$, cooled to 0 °C and 153 µl $SOCl_2$ (2.11 mmol, 2.2 equiv.) were added via syringe. After 1 h of stirring a white ppt. of **HL1**·HCl appeared. Reaction was stirred over night at rt. The white ppt. of **HL1**·HCl was isolated by filtration and washed with small amounts of $CHCl_3$ and Et_2O giving 230 mg of **HL1**·HCl. Aqueous work-up with sat. $NaHCO_3$/$CHCl_3$ yielded **HL1** (130 mg, 0.43 mmol, 45% yield). R_f (product, Cy/EE 1/1) = 0.67. Other analytical data is consistent with literature.[1]

NMR spectra

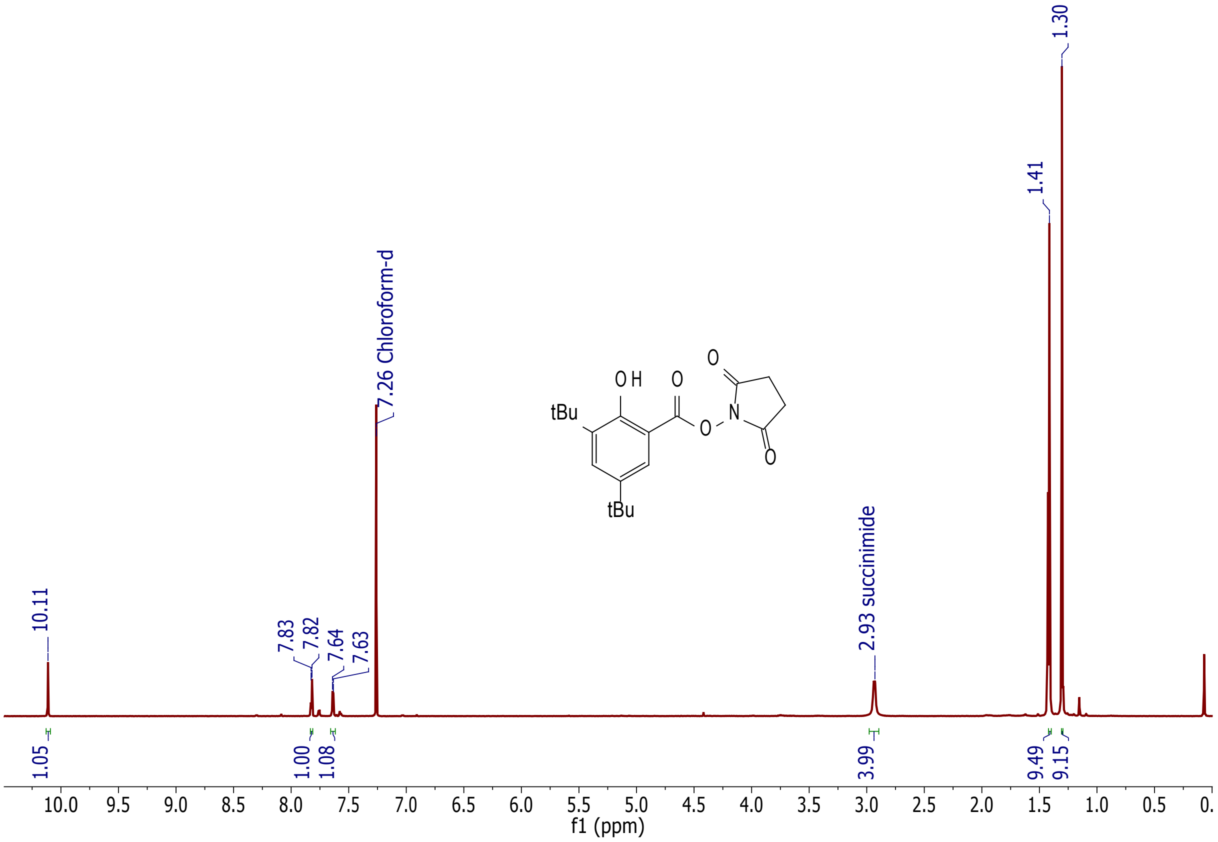

Figure S1. ^{1}H NMR spectrum of H**L1a** ($CDCl_3$).

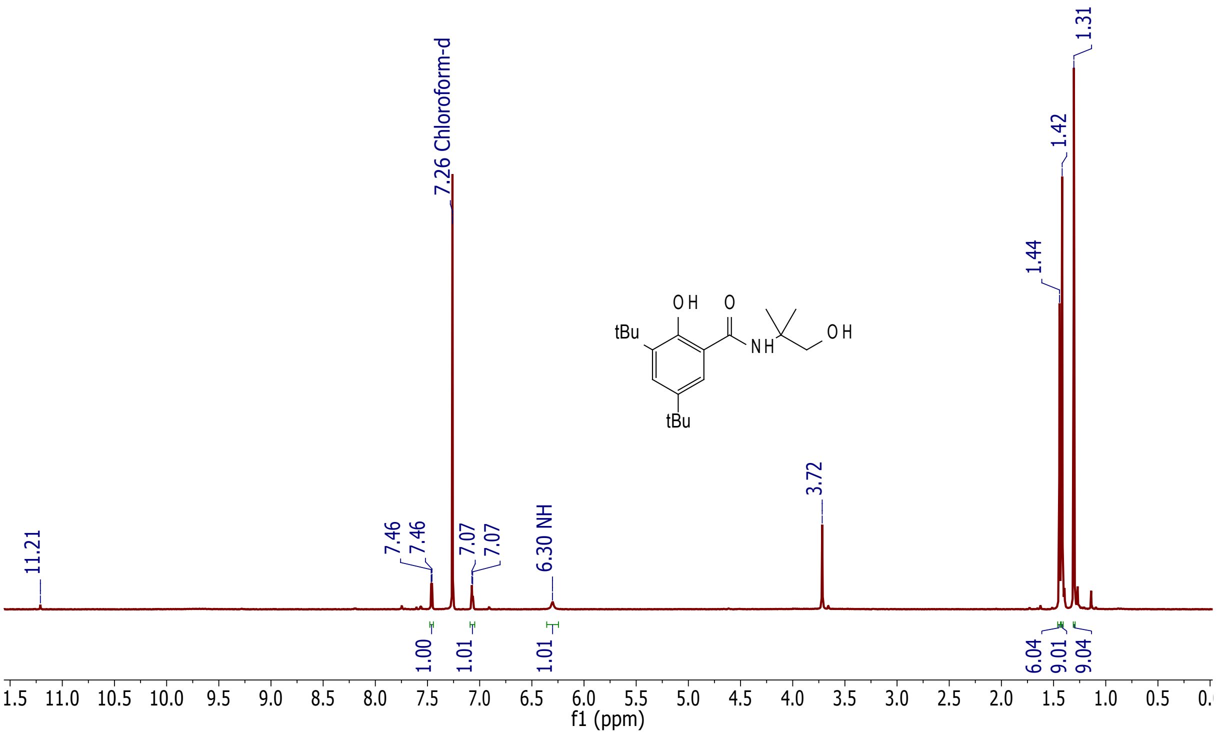

Figure S2. ^{1}H NMR spectrum of H**L1b** ($CDCl_3$).

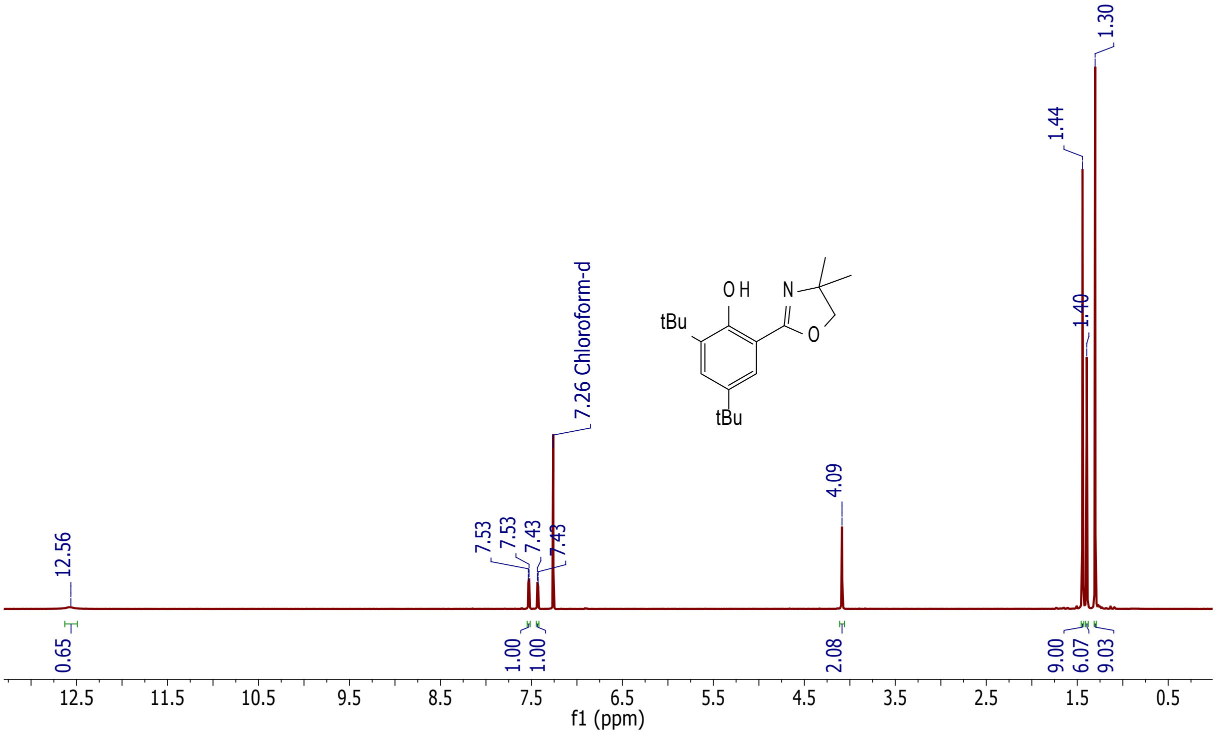

Figure S3. ^{1}H NMR spectrum of H**L1** ($CDCl_3$).

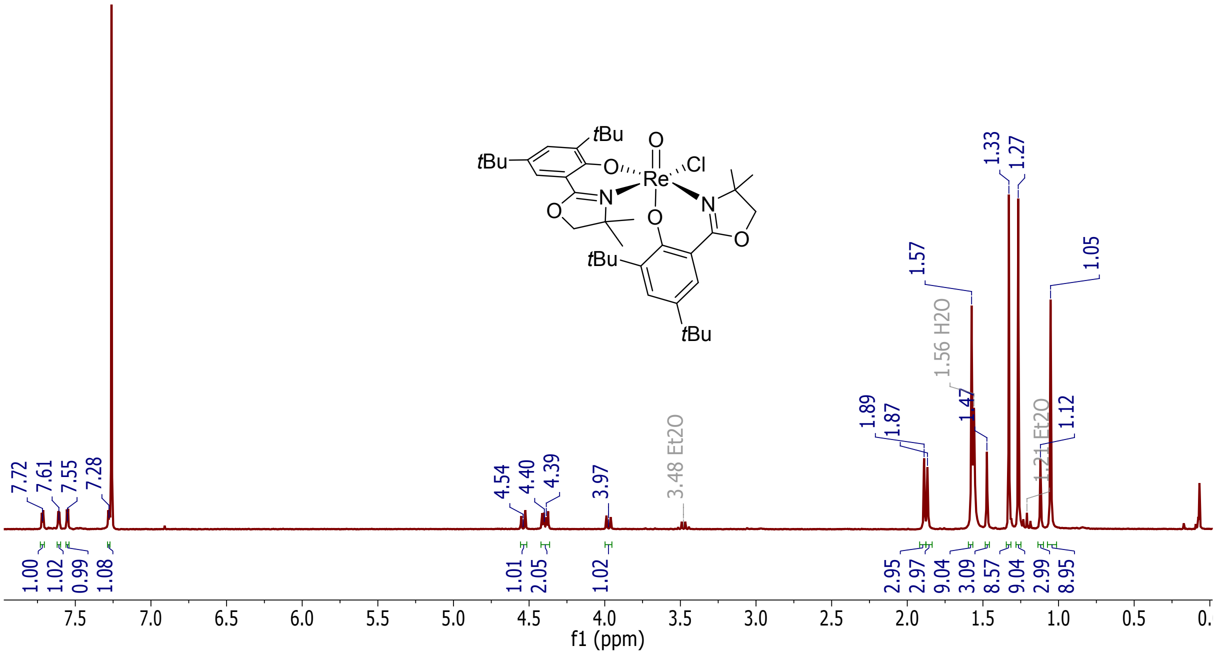

Figure S4. ^{1}H NMR spectrum of *cis*-**2** ($CDCl_3$).

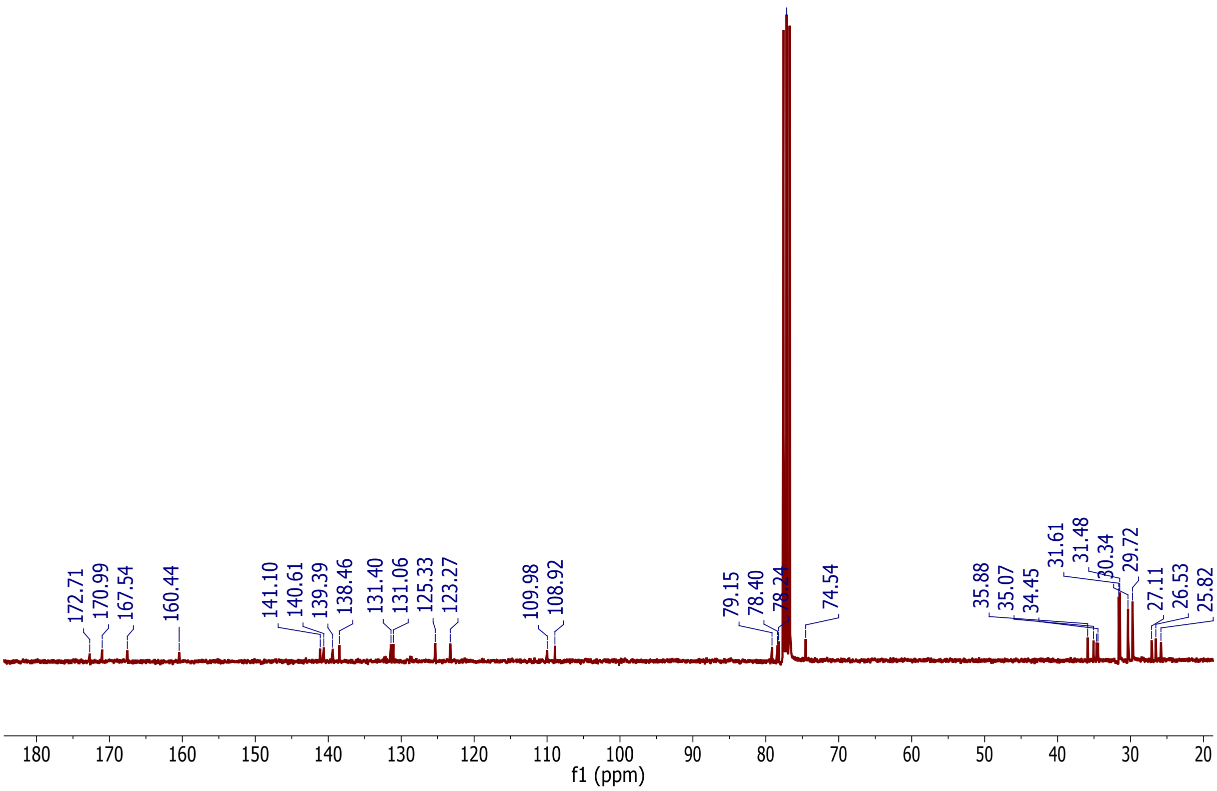

Figure S5. ^{13}C NMR spectrum of *cis*-**2** ($CDCl_3$).

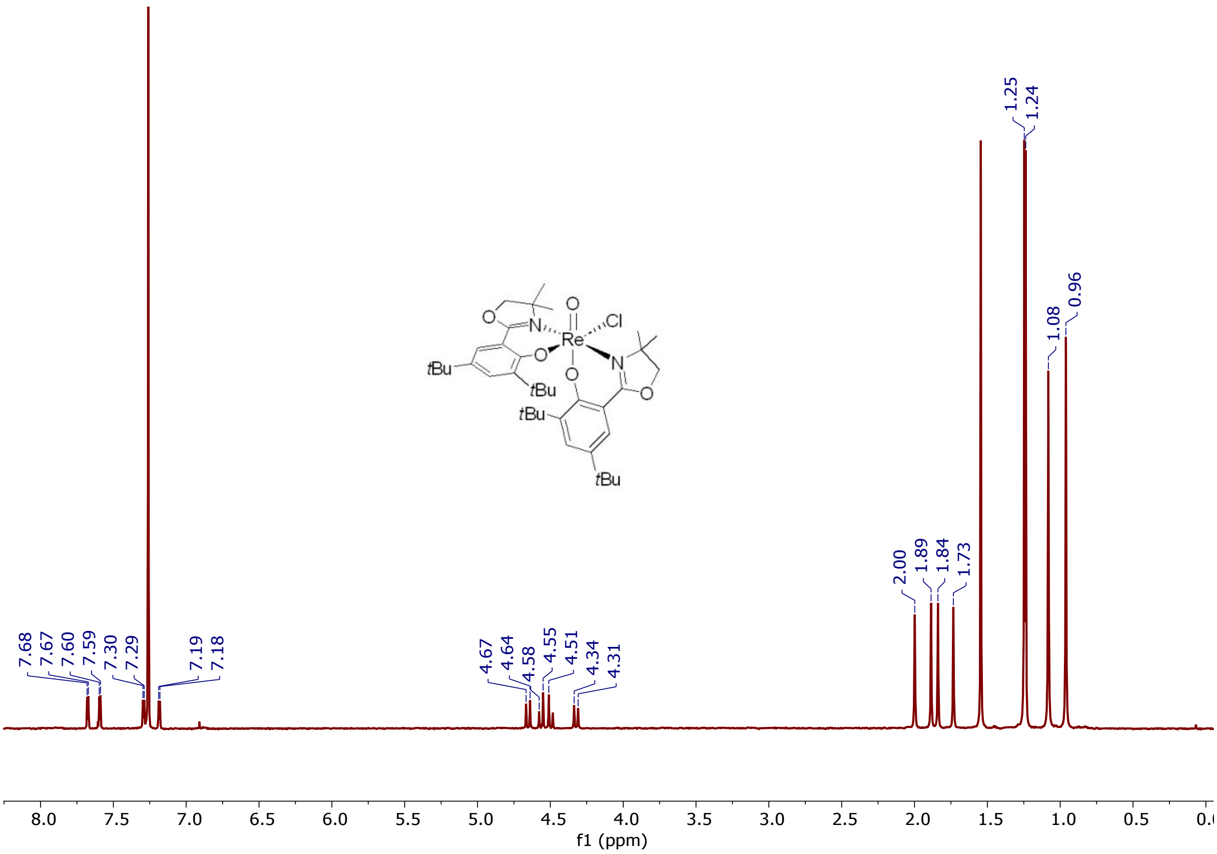

Figure S6. ^{1}H NMR spectrum of *trans*-**2** ($CDCl_3$).

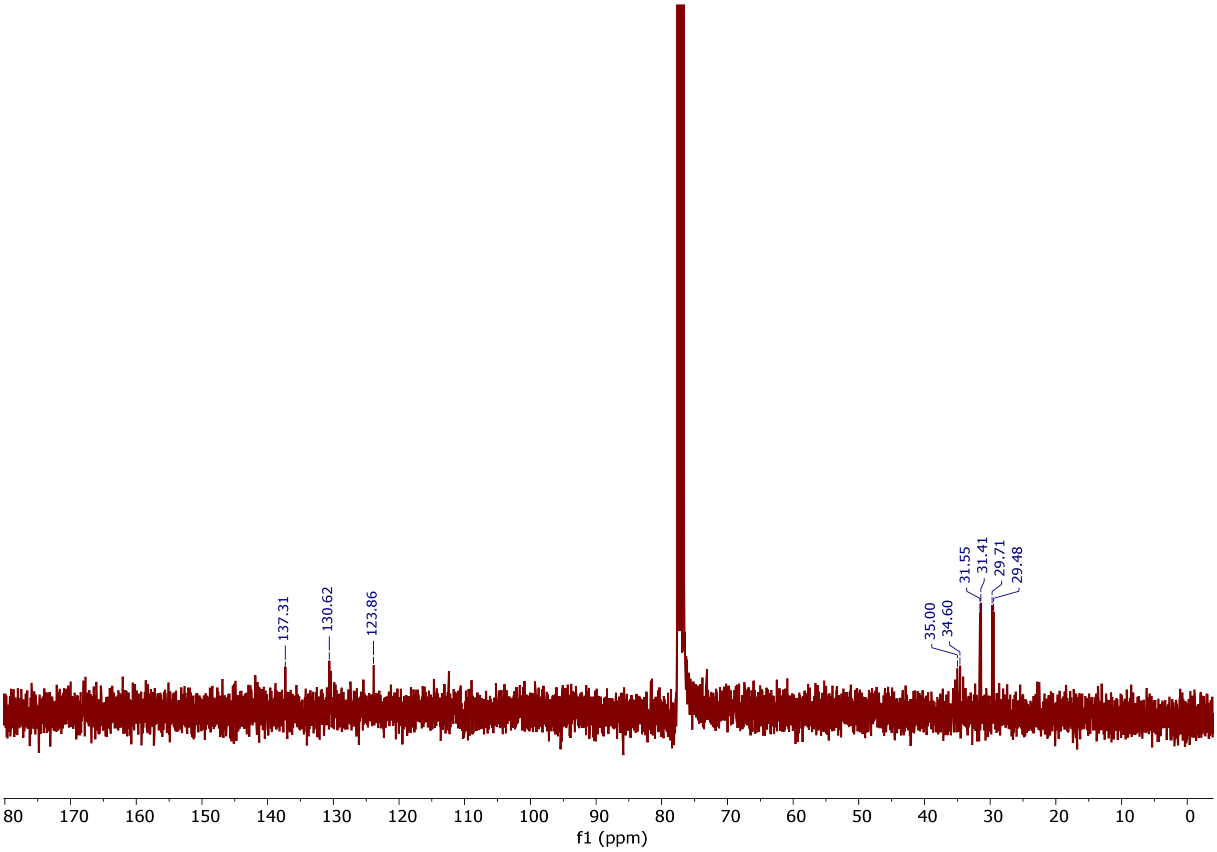

Figure S7. ^{13}C NMR spectrum of *trans*-**2** ($CDCl_3$)

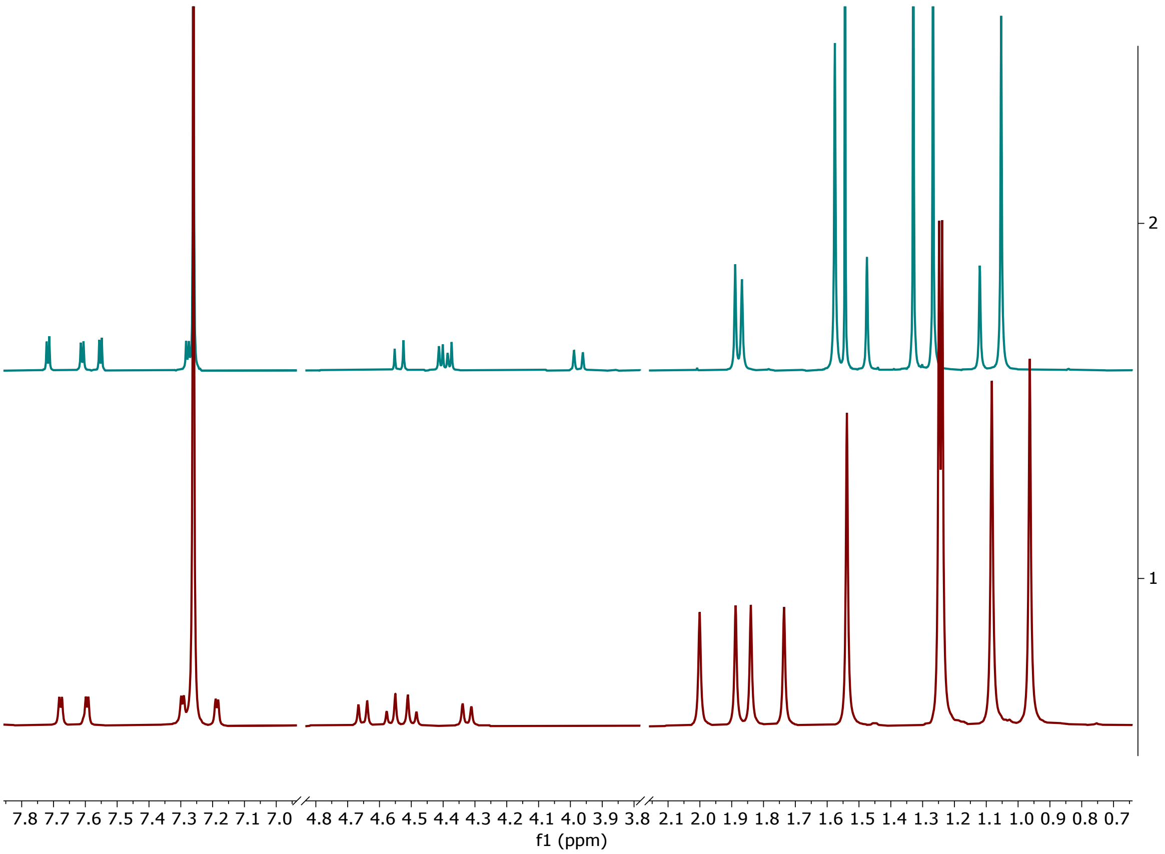

Figure S8. Comparison of ^{1}H NMR spectra of *trans*-**2** (spectrum 1, bottom) and *cis*-**2** (spectrum 2, top) ($CDCl_3$).

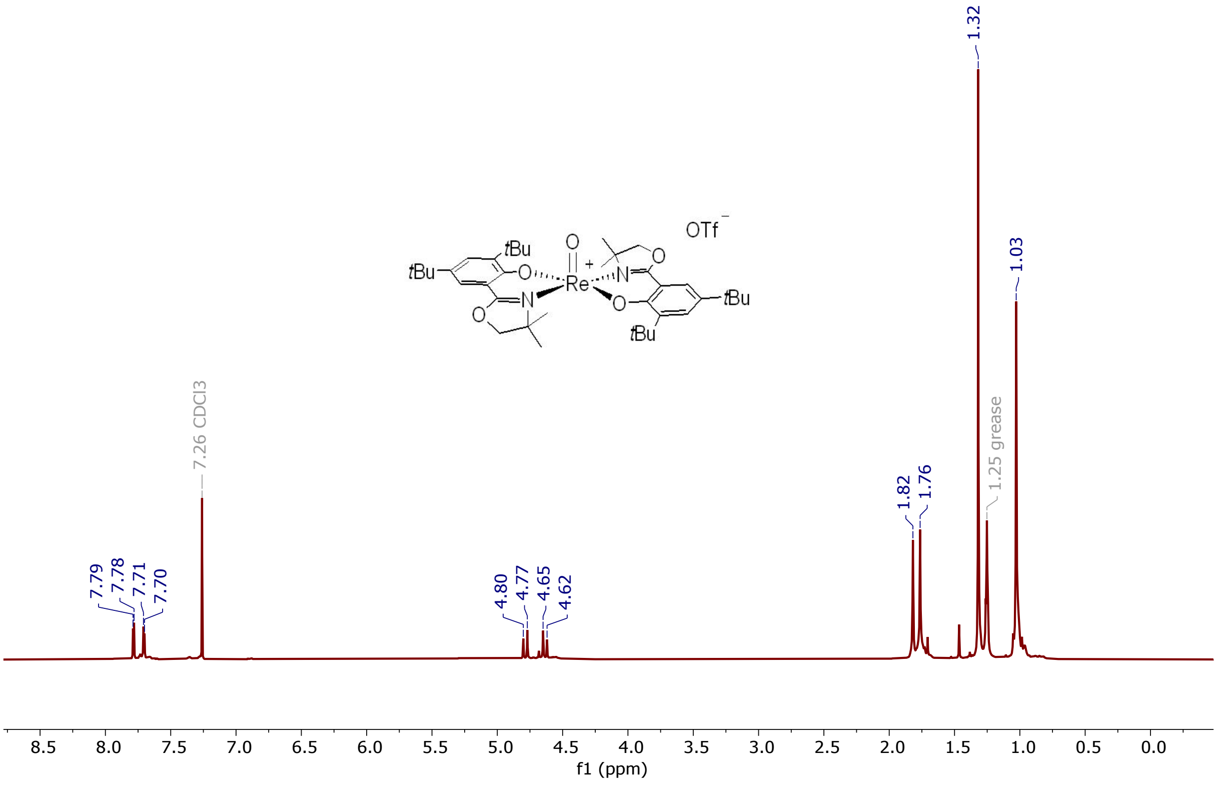

Figure S9. ^{1}H NMR spectrum of **3a** ($CDCl_3$).

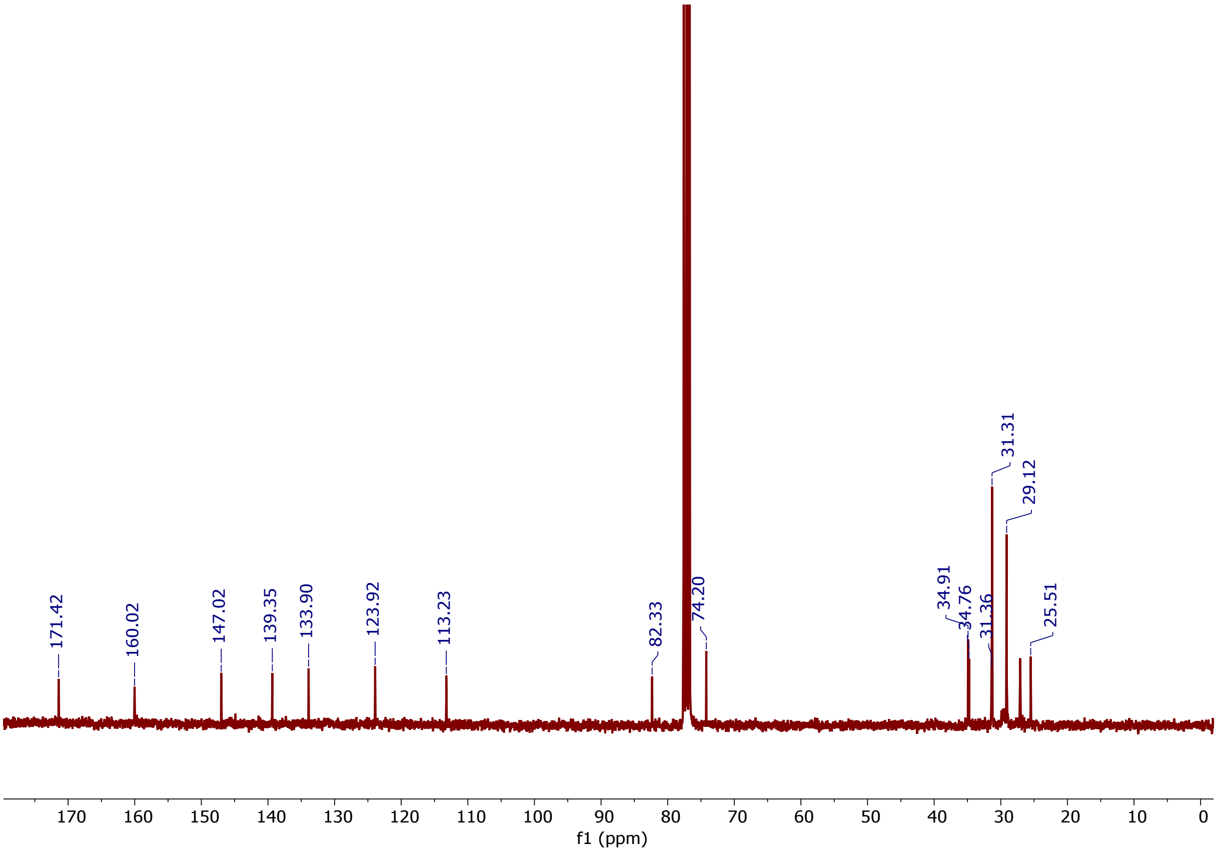

Figure S10. ^{13}C NMR spectrum of **3a** ($CDCl_3$)

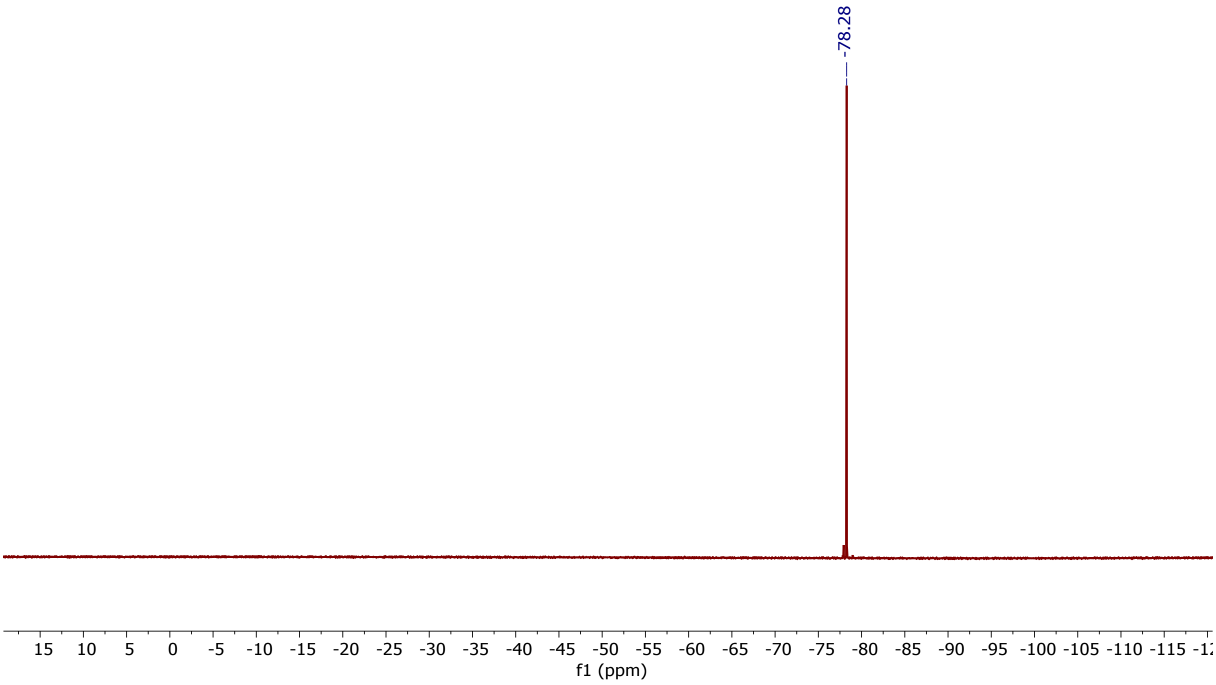

Figure S11. ^{19}F NMR spectrum of **3a** ($CDCl_3$)

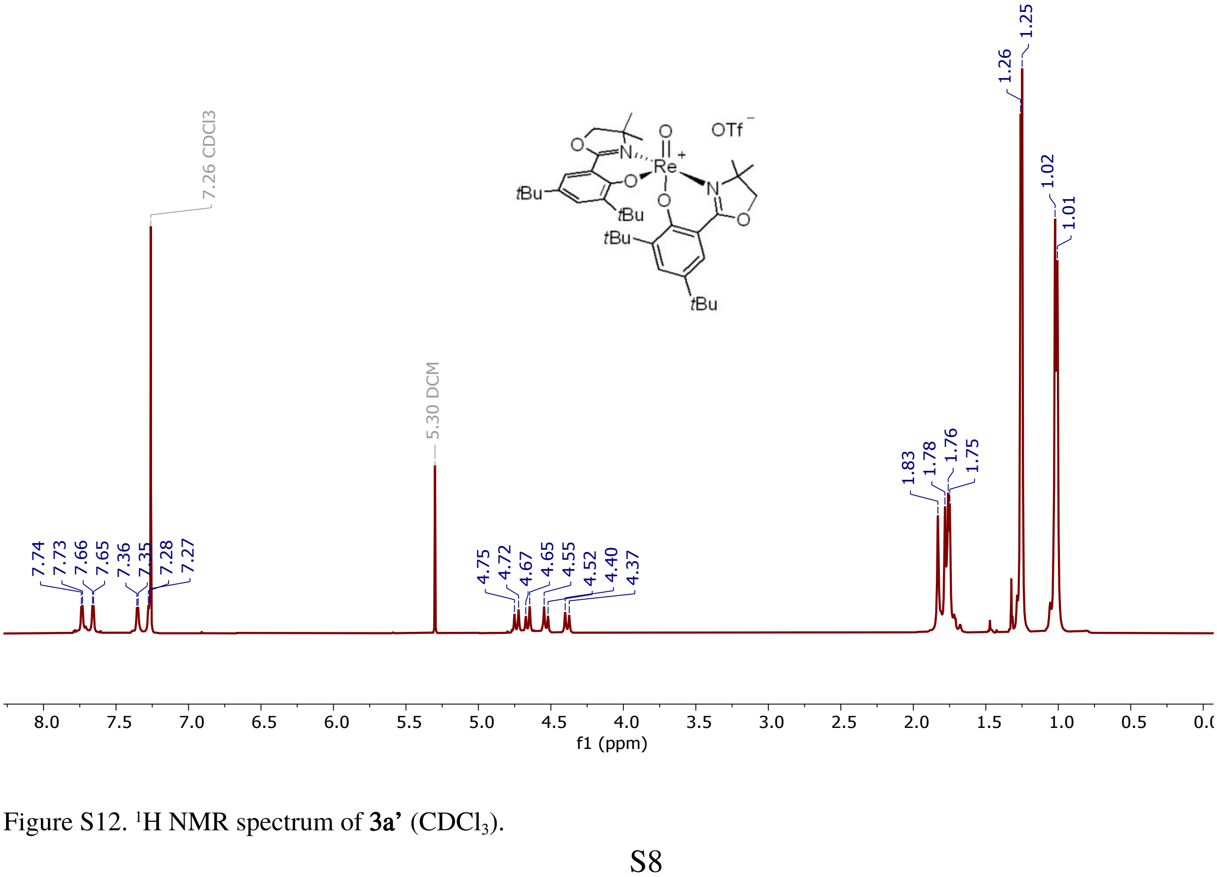

Figure S12. ^{1}H NMR spectrum of **3a’** ($CDCl_3$).

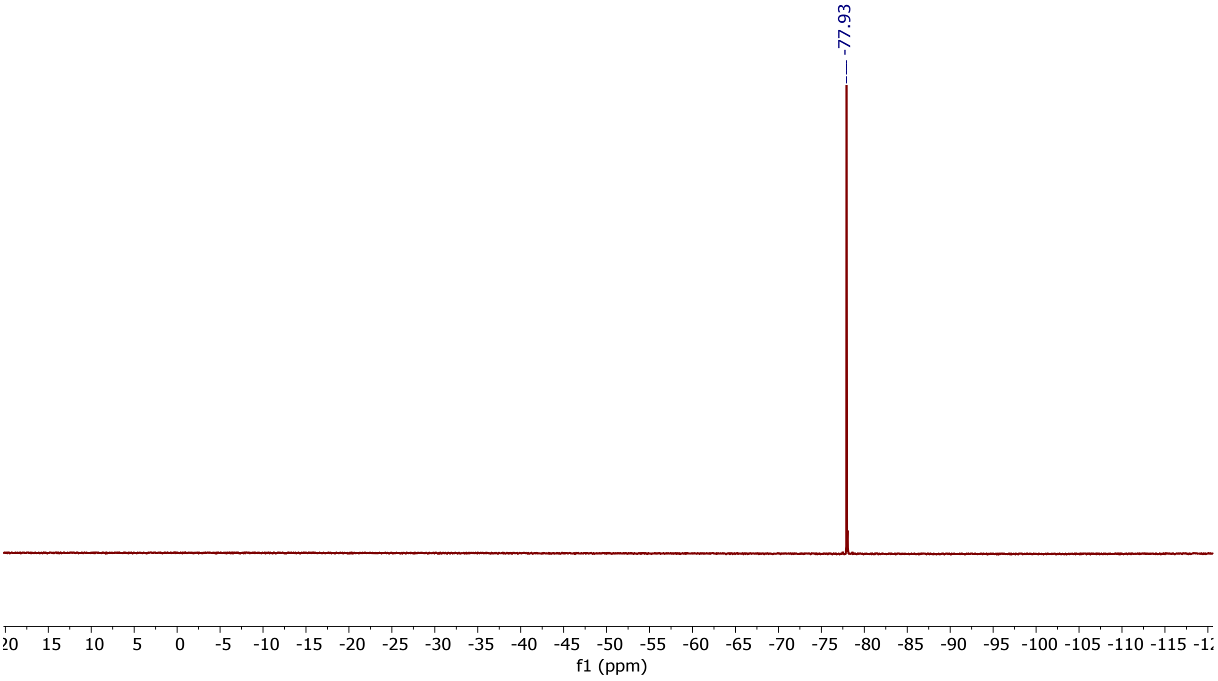

Figure S13. ^{19}F NMR spectrum of **3a’** ($CDCl_3$).

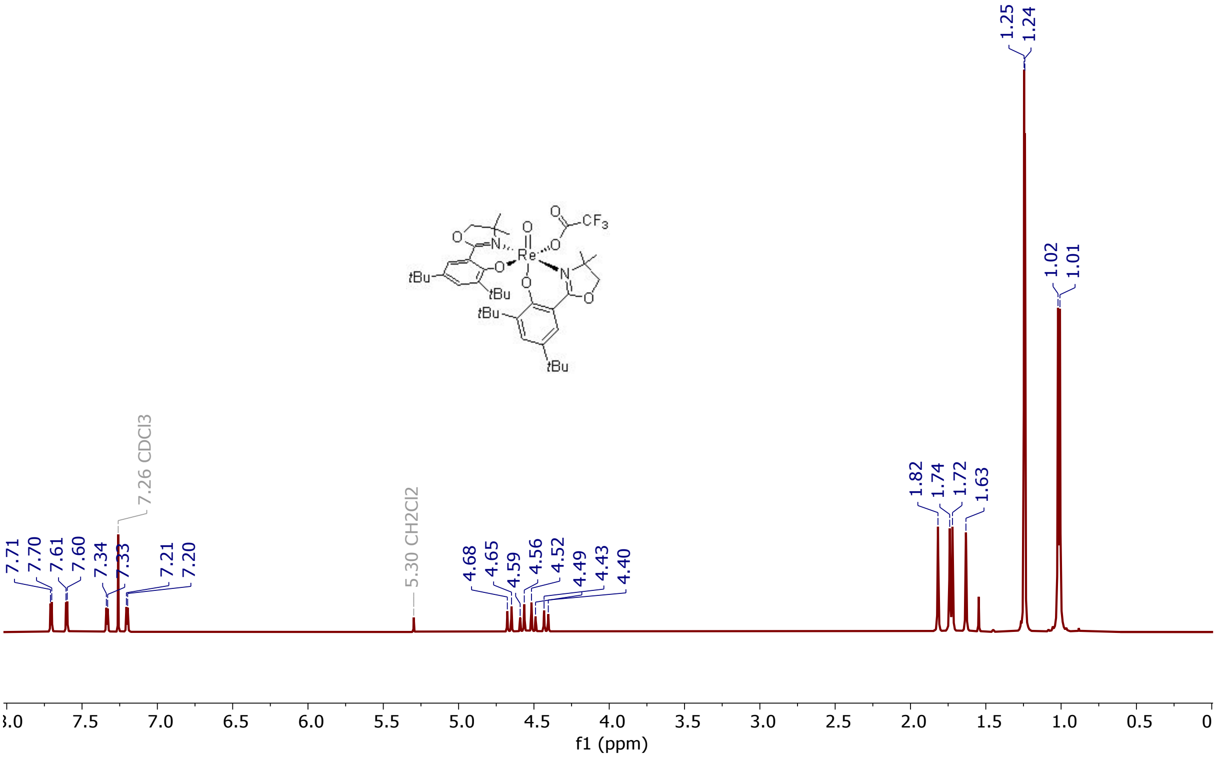

Figure S14. ^{1}H NMR spectrum of **3b** ($CDCl_3$).

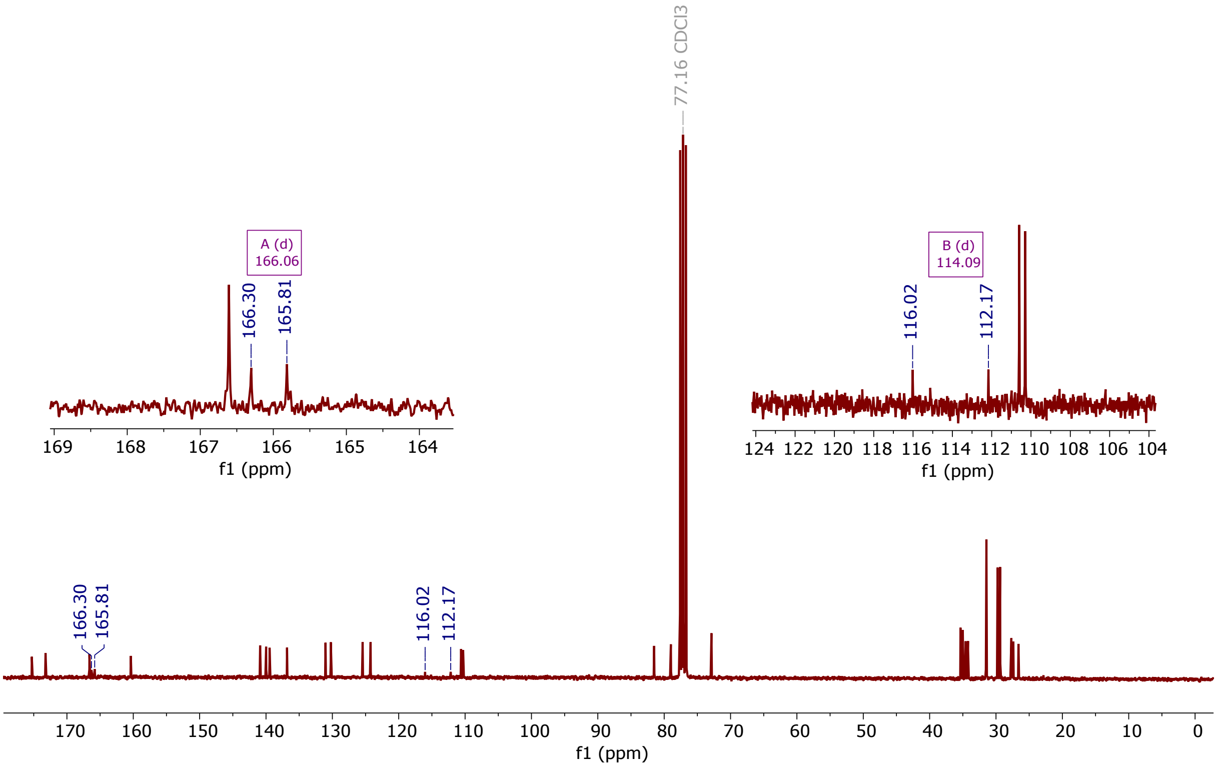

Figure S15. ^{13}C NMR spectrum of **3b**; zoom-ins show the poorly resolved q at 166.06 ppm (A, app. d, $^{2}J(^{19}F,^{13}C)$ and the poorly resolved q at 114.09 ppm (B, app. d, $^{1}J(^{19}F,^{13}C)$ of the O_2C-CF_3 anion ($CDCl_3$).

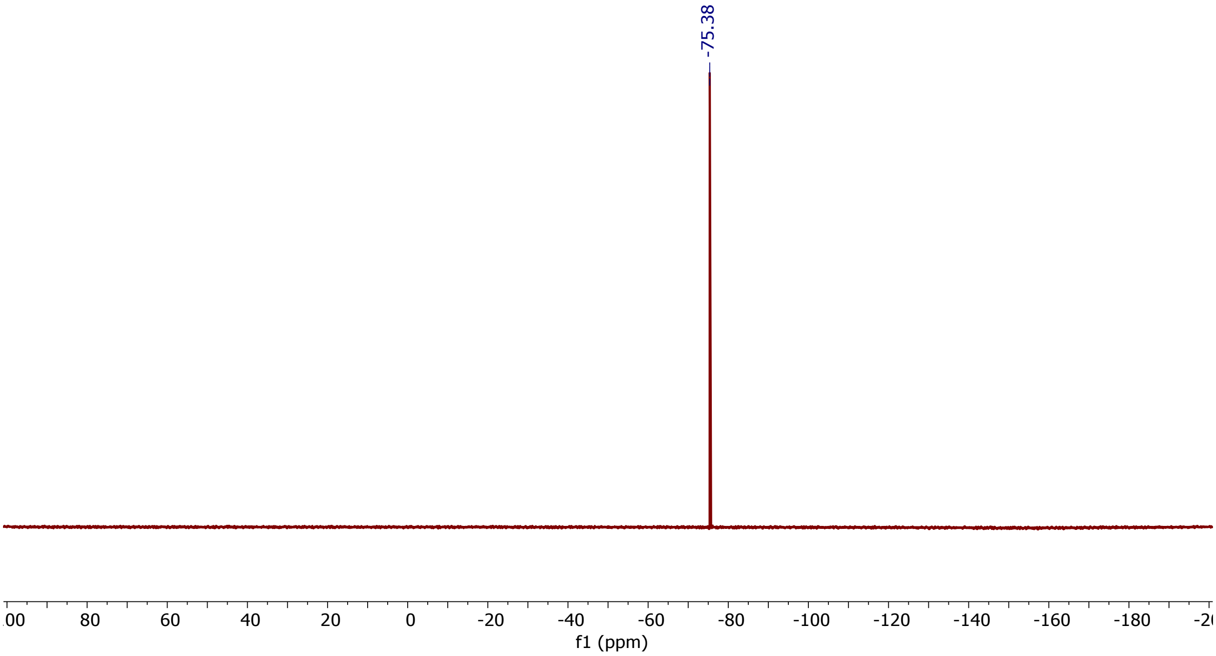

Figure S16. ^{19}F NMR spectrum of **3b** ($CDCl_3$).

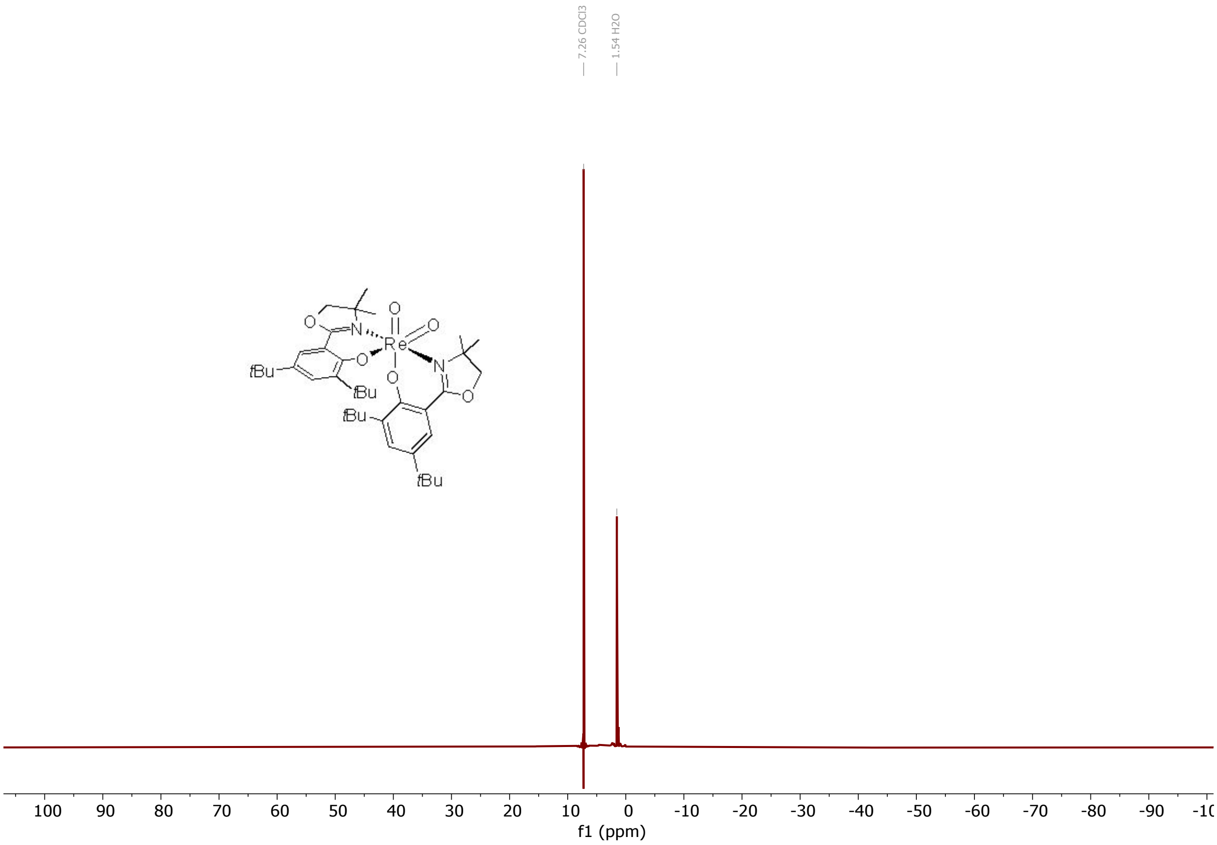

Figure S17. Extended ^{1}H NMR spectrum of paramagnetic complex **4**, showing no features ($CDCl_3$).

X-ray diffraction analysis

The X-ray data collections for complexes *cis*-**2**, *trans*-**2**, **3e** and **4** were performed with a Bruker AXS SMART APEXII CCD diffractometer at 100 K with Mo Kα radiation (λ= 0.71073 Å) from an Incoatec microfocus sealed tube equipped with a multilayer monochromator. Absorption corrections were made semi-empirically from equivalents. The structures were solved by direct methods (SHELXS-97)[3] and refined by full matrix least-squares techniques against F^2 (SHELXL-2014/6).[4] The non-hydrogen atoms were refined with anisotropic displacement parameters without any constraints. The H atoms of the CH_2 groups were refined with common isotropic displacement parameters for the H atoms of the same group and idealized geometry with approximately tetrahedral angles and C–H distances of 0.99 Å. The H atoms of the phenyl rings were put at the external bisectors of the C–C–C angles at C–H distances of 0.95 Å and common isotropic displacement parameters were refined for the H atoms of the same ring. The H atoms of the methyl groups were refined with common isotropic displacement parameters for the H atoms of the same group and idealized geometries with tetrahedral angles, enabling rotation around the C–C bonds, and C–H distances of 0.98 Å. For complex **3e**, the OH and NH hydrogen atoms were found in a difference Fourier map and refined freely. The X-ray data collections for complexes **3b** and **3d** were performed with a XtaLAB Synergy, Dualflex, HyPix-Arc 100 diffractometer at 100.0(2) K with Cu Kα radiation (λ= 1.54184 Å). Data reduction, scaling and absorption corrections were performed using the *CrysAlisPro* software.[5] A numerical absorption correction based on gaussian integration over a multifaceted crystal model and an empirical absorption correction using spherical harmonics, implemented in SCALE3 ABSPACK scaling algorithm, were performed. The structures were solved with the ShelXT 2018/2[5] solution program using the intrinsic phasing solution method and by using Olex2 1.5-ac6-020[6] as the graphical interface. The models were refined with ShelXL 2019/3[4] using full matrix least squares minimization on F^2. All non-hydrogen atoms were refined anisotropically. Hydrogen atom positions were calculated geometrically and refined using a riding model. CCDC 2362480 – 2362485 contain the supplementary crystallographic data for this paper. This data can be obtained free of charge via

http://www.ccdc.cam.ac.uk/ or from Cambridge Crystallographic Data Centre, 12 Union Road, Cambridge, CB2 1EZ, UK.

For complex *trans*-**2**, since twinning was detected, an appropriate twin matrix (-1 0 0 / 0 -1 0 / 0 0 -1) was applied with a BASF factor of 0.344(2) between the two unequal twin components. The asymmetric unit consists of two complexes A and B. In complex B, the chloro-oxo-rhenium(V) group was disordered over two orientations which refined to site occupation factors of 0.806(8) and 0.194(8), respectively. EADP and SAME restraints were used to model this disorder. For complex **3e**, one of the four perrhenate groups of the asymmetric unit was disordered over two orientations which refined to site occupation factors of 0.658(3) and 0.342(3), respectively. At the disordered group, the same anisotropic displacement parameters were used for equivalent atoms and the Re–O distances were fixed to 1.71 Å. For complex **4**, the *tert*-butyl group bonded to C24 was disordered over two orientations which refined to site occupation factors of 0.680(8) and 0.320(8), respectively and EADP and DFIX restraints were used.

Table S1. Crystal data and structure refinement for *cis*-**2**, *trans*-**2** and **3b**.

Compound	*cis*-**2**	*trans*-**2**	**3b**
CCDC n°	1913786	2214693	2468512
Identification code	JS75B	JSCR5C	AD163
Empirical formula	$C_{38}H_{56}ClN_2O_5Re$	$C_{38}H_{56}ClN_2O_5Re$	6 [$C_{38}H_{56}N_2O_9Re_2$] • 6 $CHCl_3$ • C_6H_{12}
Formula weight	842.49	842.49	7143.83
Crystal system	triclinic	monoclinic	trigonal
Space group	P-1	$P2_1$	R-3
a /Å	9.6111(14)	11.1949(8)	39.1305(2)
b /Å	13.378(2)	11.8739(9)	39.1305(2)
c /Å	16.476(2)	29.1915(19)	15.39490(10)
α /°	103.124(3)	90	90
β /°	106.858(3)	99.750(4)	90
γ /°	97.963(4)	90	120
Volume /$Å^3$	1926.4(5)	3824.3(5)	20414.5(2)
Z	2	4	3
ρ_{calc} g/cm^3	1.452	1.463	1.743
μ /mm^{-1}	3.265	3.290	12.330
F(000)	860.0	1720.0	10548.0
Crystal size /mm^3	0.17 × 0.12 × 0.11	0.11× 0.06 × 0.03	0.24 × 0.2 × 0.2
Radiation	Mo Kα (λ=0.71073 Å)	Mo Kα (λ=0.71073 Å)	Cu Kα (λ = 1.54184 Å)
2Θ range for data collection/°	3.20 to 60.00	1.42 to 52.00	6.306 to 154.738
Index ranges	$-13 \leq h \leq 13$, $-18 \leq k \leq 18$, $-23 \leq l \leq 23$	$-13 \leq h \leq 13$, $-13 \leq k \leq 14$, $-35 \leq l \leq 35$	$-49 \leq h \leq 48$, $-47 \leq k \leq 47$, $-19 \leq l \leq 18$
Reflections collected	50906	47280	50512
Independent reflections	9895 [R_{int} = 0.0664, R_{sigma} = 0.0575]	10258 [R_{int} =0.1070, R_{sigma}= 0.1566]	9615 [R_{int} = 0.0422, R_{sigma} = 0.0286]
Data / restraints / parameters	11230 / 0 /460	13224 / 77 / 878	9615 / 0 / 521
Goodness-of-fit on F^2	1.027	1.038	1.114
Final R indexes [I ≥ 2σ(I)]	R_1 = 0.0288 wR_2 = 0.0572	R_1 = 0.0675, wR_2 = 0.1334	R_1 = 0.0299, wR_2 = 0.0679
Final R indexes [all data]	R_1 = 0.0374 wR_2 = 0.0598	R_1 = 0.0892 wR_2 = 0.1428	R_1 = 0.0310, wR_2 = 0.0685

Largest diff. peak/hole / e.Å^{-3}	1.222 / -1.357	1.885 / -1.628	2.36 / -1.83

Table S2. Crystal data and structure refinement for **3d**, **3e** and **4**.

Compound	**3d**	**3e**	**4**
CCDC n°	2468513	2065055	2214694
Identification code	AD176	JS75Z	JSCR13K
Empirical formula	$C_{40}H_{56}F_3N_2O_7Re$	$C_{19}H_{30}NO_2^{+}ReO_4^{-}$	$C_{38}H_{56}N_2O_6Re$
Formula weight	920.06	544.66	823.04
Crystal system	orthorhombic	triclinic	monoclinic
Space group	Pbca	P-1	$P2_1/c$
a /Å	11.57980(10)	9.3218(11)	11.0684(8)
b /Å	25.1347(2)	18.795(2)	11.3563(8)
c /Å	28.7333(3)	25.892(3)	30.152(2)
α /°	90	76.581(9)	90
β /°	90	79.686(7)	100.428(3)
γ /°	90	89.767(7)	90
Volume /Å^3	8362.97(13)	4337.9(9)	3727.4(5)
Z	8	8	4
ρ_{calc} g/cm^3	1.461	1.699	1.467
μ /mm^{-1}	6.199	5.634	3.306
F(000)	3744.0	2192.0	1684.0
Crystal size /mm^3	0.16 × 0.10 × 0.09	0.34× 0.12 × 0.03	0.13 × 0.04 × 0.03
Radiation	Cu Kα (λ = 1.54184 Å)	Mo Kα (λ=0.71073 Å)	Mo Kα (λ=0.71073 Å)
2Θ range for data collection/°	6.152 to 149.004	4.38 to 56.00	4.22 to 60.00
Index ranges	$-14 \le h \le 13$, $-31 \le k \le 26$, $-35 \le l \le 33$	$-12 \le h \le 12$, $-24 \le k \le 24$, $-34 \le l \le 34$	$-13 \le h \le 13$, $-13 \le k \le 13$, $-35 \le l \le 35$
Reflections collected	48426	31565	97559
Independent reflections	8533 [R_{int} = 0.0346, R_{sigma} = 0.0252]	14219 [R_{int} =0.0490, R_{sigma}= 0.1222]	5218 [R_{int} = 0.1134, R_{sigma} = 0.0846]
Data / restraints / parameters	8533 / 0 /522	20969 / 22 / 1071	6556 / 8 / 476
Goodness-of-fit on F^2	1.257	1.011	1.095
Final R indexes [I ≥ 2σ(I)]	R_1 = 0.0418, wR_2 = 0.0906	R_1 = 0.0564, wR_2 = 0.1328	R_1 = 0.0413, wR_2 = 0.0846

Final R indexes [all data]	$R_1 = 0.0448$, $wR_2 = 0.0916$	$R_1 = 0.0947$ $wR_2 = 0.1475$	$R_1 = 0.0597$, $wR_2 = 0.0923$
Largest diff. peak/hole / e.Å^{-3}	1.04 / -1.83	1.646 / -1.683	1.692 / -1.385

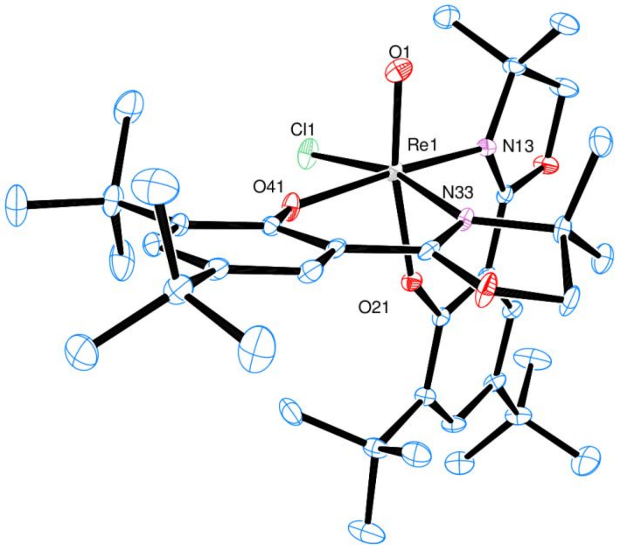

Figure S18. Molecular structure of *cis*-**2**. The probability ellipsoids are drawn at 50% level. Protons are omitted for clarity.

Table S3. Selected bond lengths (Å) and angles(°) for *cis*-**2**.

Re1-O1	1.6844(18)
Re1-O21	1.9630(17)
Re1-O41	1.9800(16)
Re1-N13	2.197(2)
Re1-N33	2.131(2)
Re1-Cl1	2.3876(7)
O1-Re1-O21	168.41(7)
N13-Re1-O41	165.55(7)
N33-Re1-Cl1	166.02(6)
C12-N13-C14	107.7(2)

C12-N13-Re1	125.18(16)
C14-N13-Re1	124.68(16)
C21-O21-Re1	141.52(15)
C32-N33-C34	106.7(2)
C32-N33-Re1	122.79(17)
C34-N33-Re1	130.49(15)
C41-O41-Re1	132.03(15)

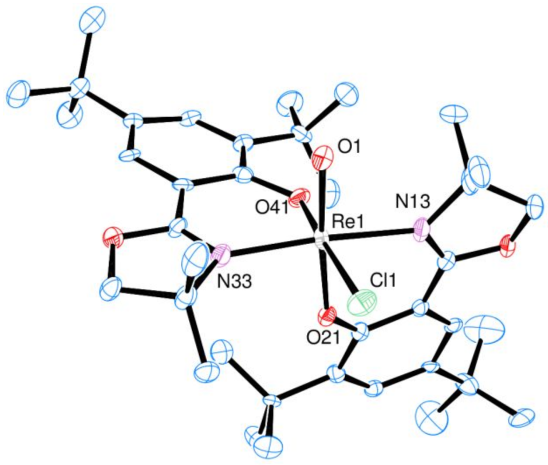

Figure S19. Molecular structure of *trans*-**2**. The probability ellipsoids are drawn at 50% level. Only complex A of the asymmetric unit cell is shown. Protons are omitted for clarity.

Table S4. Selected bond lengths (Å) and angles(°) for *trans*-**2**.

Re1-O1	1.857(12)
Re1-O21	1.967(11)
Re1-O41	1.980(11
Re1-N13	2.135(12)
Re1-N33	2.077(13)
Re1-Cl1	2.325(5)
Re2-O2	1.856(17)
Re2-O61	1.979(14)
Re2-O81	1.949(14)
Re2-N53	2.119(14)
Re2-N73	2.072(14)
Re2-Cl2	2.317(7)
O1-Re1-O21	174.9(5)
N13-Re1-N33	173.2(5)
O41-Re1-Cl1	170.6(3)
C12-N13-C14	108.5(12)
C12-N13-Re1	126.0(11)
C14-N13-Re1	125.2(8)

C21-O21-Re1	135.9(10
C32-N33-C34	108.9(12)
C32-N33-Re1	122.4(11)
C34-N33-Re1	128.4(10)
C41-O41-Re1	128.0(9)
O2-Re2-O61	176(3)
N53-Re2-N73	172.5(7)
O81-Re2-Cl2	169.3(5)
C52-N53-C54	107.1(14)
C52-N53-Re3	119.0(13
C54-N53-Re3	130.6(12)
C61-O61-Re3	127.5(11)
C72-N73-C74	108.5(14)
C72-N73-Re2	122.8(13)
C74-N73-Re2	128.6(10)
C81-O81-Re2	126.8(12)

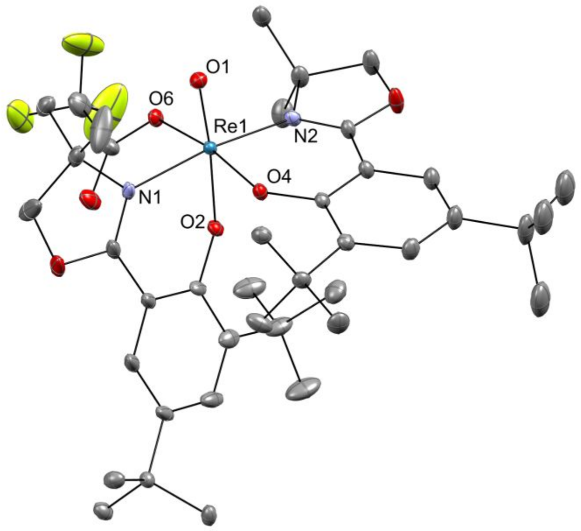

Figure S20. Molecular structure of **3b**. The probability ellipsoids are drawn at 30% level. The H atoms were omitted for clarity. For the disordered tert-butyl group, only the positions with the highest occupancies are depicted.

Table S5. Selected bond lengths (Å) and angles (˚) for **3b**.

Re1-O1	1.677(3)
Re1-O2	1.958(3)
Re1-O4	2.012(3)
Re1-O6	2.088(3)
Re1-N1	2.130(4)
Re1-N2	2.083(4)
O2-C1	1.341(5)
O4-C21	1.333(5)
N1-C7	1.284(6)
N2-C27	1.304(5)
O1-Re1-O2	174.90(15)
O1-Re1-O4	96.45(15)
O1-Re1-O6	92.85(15)
O1-Re1-N1	92.39(16)
O1-Re1-N2	95.20(16)
O2-Re1-O4	85.41(13)
O2-Re1-O6	85.08(13)
O2-Re1-N1	82.95(15)
O2-Re1-N2	89.52(14)
O4-Re1-O6	170.22(13)
O4-Re1-N1	87.05(14)
O4-Re1-N2	90.77(13)
O6-Re1-N1	89.56(14)
N2-Re1-O6	91.40(14)
N2-Re1-N1	172.30(16)
C1-O2-Re1	136.5(3)
C21-O4-Re1	129.2(3)
C7-N1-Re1	125.7(4)
C27-N2-Re1	122.1(3)

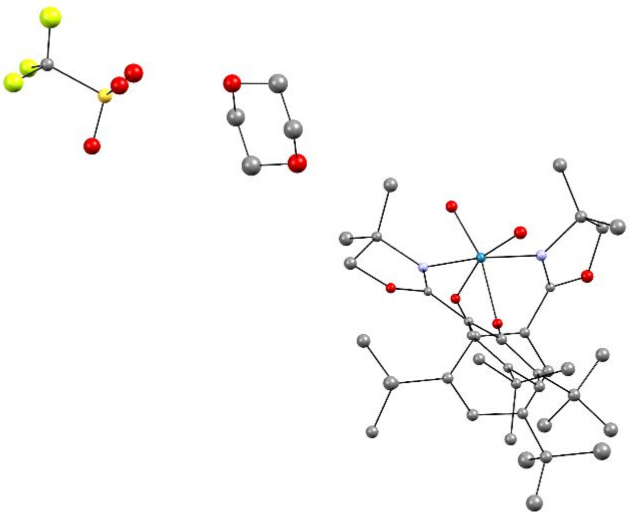

Figure S21. Molecular connectivity of complex $[ReO_2(\mathbf{L1})_2]OTf$ (**3c**). Complex **3c** was isolated as a dioxane adduct from a crystallization experiment of $[ReO(\mathbf{L1})_2]OTf$ (**3a**) in CH_2Cl_2/dioxane. A full solution of the diffraction data was not possible due to low quality of the diffraction of the crystal. The exact conditions of formation of **3c** are unclear.

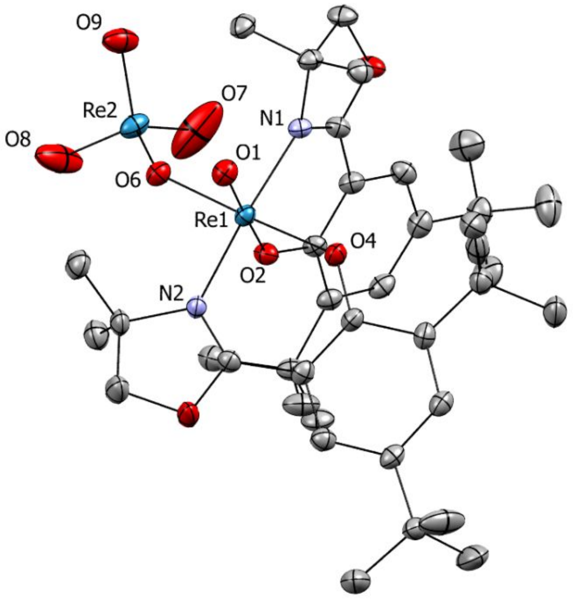

Figure S22. Molecular structure of **3d**. The probability ellipsoids are drawn at 50% level. The H atoms and solvent molecules were omitted for clarity

Table S6. Selected bond lengths (Å) and angles (°) for **3d**.

Re1-O1	1.688(3)
Re1-O2	1.970(2)
Re1-O4	1.983(2)
Re1-O6	2.108(2)
Re1-N1	2.133(3)
Re1-N2	2.066(3)
Re2-O6	1.766(2)
Re2-O7	1.689(4)
Re2-O8	1.706(4)
Re2-O9	1.709(3)
O2-C1	1.340(4)
O4-C21	1.343(4)
N1-C7	1.294(5)
N1-C9	1.514(4)
N2-C27	1.304(5)
N2-C29	1.507(4)
O1-Re1-O2	172.53(11)
O1-Re1-O4	98.14(11)
O1-Re1-O6	91.95(11)
O1-Re1-N1	92.16(12)
O1-Re1-N2	95.13(12)
O2-Re1-O4	86.62(10)
O2-Re1-O6	82.76(10)

O2-Re1-N1	82.18(10)
O2-Re1-N2	90.61(11)
O4-Re1-O6	168.24(10)
O4-Re1-N1	88.32(10)
O4-Re1-N2	89.91(10)
O6-Re1-N1	85.27(10)
N2-Re1-O6	95.22(10)
N2-Re1-N1	172.67(11)
O7-Re2-O6	110.95(15)
O7-Re2-O8	109.5(3)
O7-Re2-O9	108.9(2)
O8-Re2-O6	109.26(17)
O8-Re2-O9	107.84(18)
O9-Re2-O6	110.36(13)
C1-O2-Re1	137.7(2)
C21-O4-Re1	129.9(2)
Re2-O6-Re1	142.46(14)
C7-N1-Re1	126.1(2)
C9-N1-Re1	125.2(2)
C27-N2-Re1	123.8(2)
C29-N2-Re1	127.6(2)

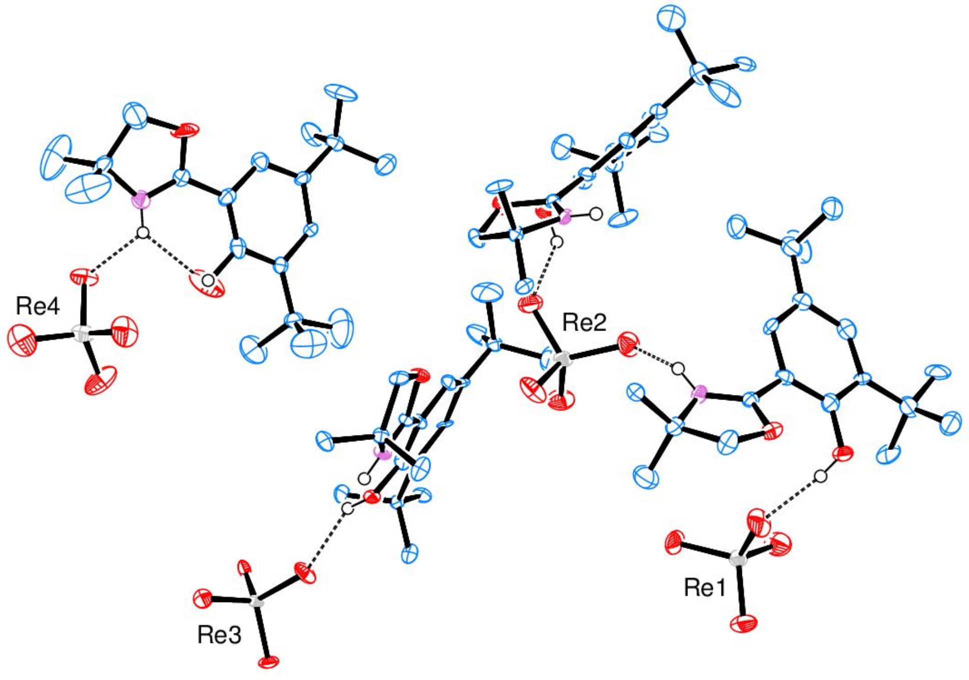

Figure S23. Molecular structure of **3e**. The probability ellipsoids are drawn at 50% level. Disordered perrhenate anion of the asymmetric unit cell with lower occupancy is not shown. Protons except the O-H and N-H are omitted for clarity. H-bonds are shown with dashed lines.

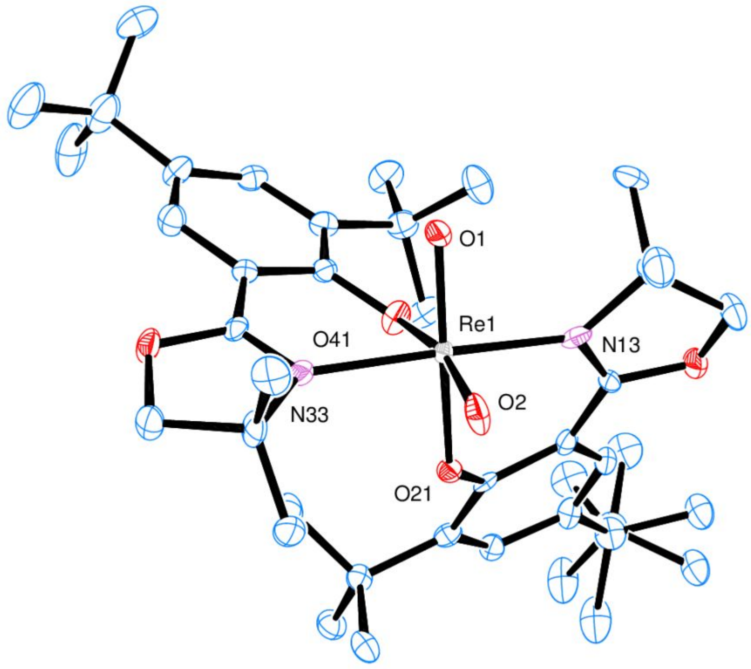

Figure S24. Molecular structure of **4**. The probability ellipsoids are drawn at 50% level. Disordered atoms of the asymmetric unit cell with lower occupancy are not shown. Protons are omitted for clarity.

Table S7. Selected bond lengths (Å) and angles(°) for **4**.

Re1-O1	1.745(4)
Re1-O2	1.726(4)
Re1-O21	2.046(4)
Re1-O41	2.062(4)
Re1-N13	2.079(6)
Re1-N33	2.112(6)
O21-C21	1.328(7)
O41-C41	1.348(8)
O1-Re1-O21	162.5(2)
O2-Re1-O41	164.42(19)
N13-Re1-N33	173.5(2)

C12-N13-C14	106.7(6)
C12-N13-Re1	126.0(5)
C14-N13-Re1	127.3(4)
C21-O21-Re1	128.6(4)
C32-N33-C34	109.0(6)
C32-N33-Re1	122.7(4)
C34-N33-Re1	127.7(4)
C41-O41-Re1	127.1(4)

DFT calculations

All geometries were optimized using the r^{2}SCAN functional in combination with the D4 dispersion correction. Frequency calculations were performed to ensure that the optimization has converged to a stationary point. Energy differences for the isomers 2A-F were calculated at the optimized geometries using the PBE0[8] functional in combination with the D4 dispersion correction[9] and the TMHF functional.[10] The def2-TZVPP basis set was used in all calculations.[11] For Re, an effective core potential (ECP) describing 60 core electrons was used.[12] DFT calculations were converged to changes 10^{-8} a.u. in changes of the energy and 10^{-7} a.u. for changes in the density matrix. All calculations were performed using a development version of Turbomole V7.9.[13] The optimized PBE0-D4 geometries for each monomer are shown in Figs. S22 to S28. XYZ files for each optimized geometry are attached as machine readable ASCII files.

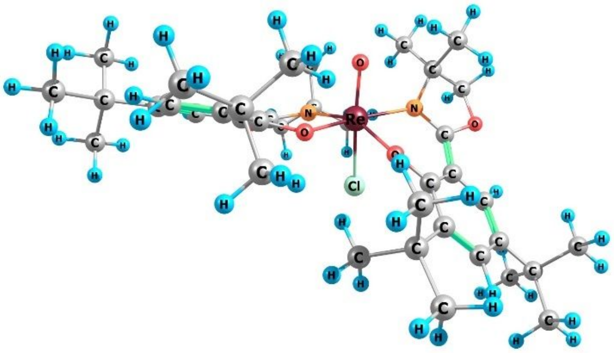

Figure S25. Calculated structure of symmetric N,N-*cis* isomer A.

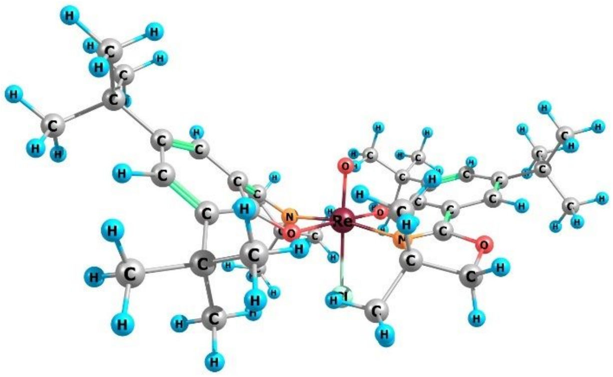

Figure S26. Calculated structure of symmetric N,N-*trans* isomer B.

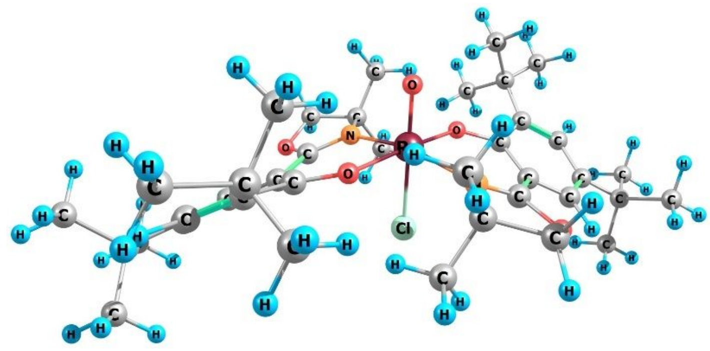

Figure S27. Calculated structure of symmetric N,N-*trans* isomer B2

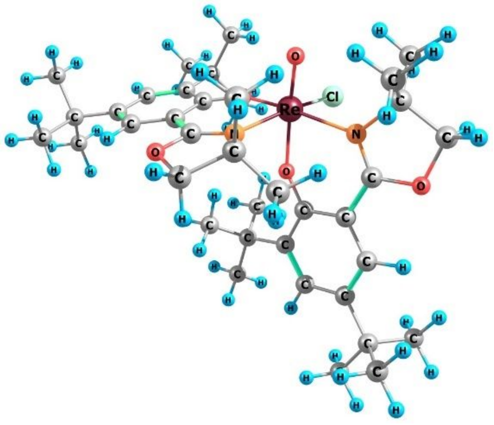

Figure S28. Calculated structure of N,N-*cis* isomer C.

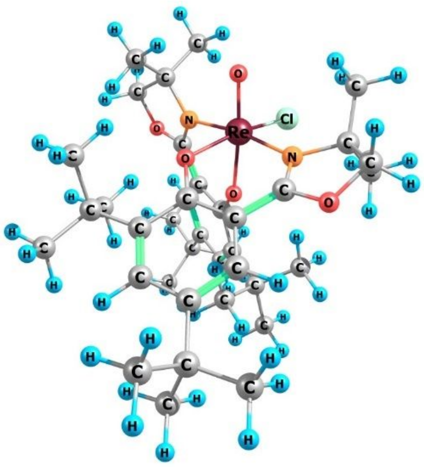

Figure S29. Calculated structure of N,N-*trans* isomer D.

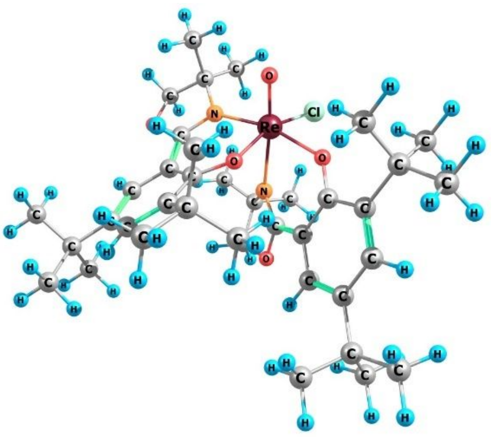

Figure S30. Calculated structure of O,O-*cis* isomer E.

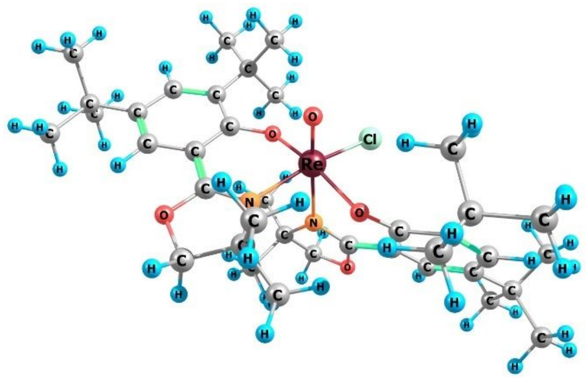

Figure S31. Calculated structure of O,O-*trans* isomer F.

References

[1] Bott, RKJ; Hammond, M; Horton, PN; Lancaster, Simon J.; Bochmann, Manfred; Scott, Peter. Group 4 salicyloxazolines are potent polymerization catalysts. *Dalton Trans.* **2005**, *34*, 3611–3613. 10.1039/b509807f.

[2] Jiménez, CA; Belmar, JB. Synthesis of highly hindered polyanionic chelating ligands. *Tetrahedron* **2005**, *61*, 3933–3938. 10.1016/j.tet.2005.02.064.

[3] Sheldrick, GM. A short history of SHELX. *Acta Crystallogr., Sect. A: Found.* **2008**, 112–122. 10.1107/s0108767307043930.

[4] Sheldrick, GM. Crystal structure refinement with SHELXL. *Acta Crystallogr., Sect. C: Cryst. Struct. Chem.* **2015**, 3–8. 10.1107/S2053229614024218.

[5] Sheldrick, GM. SHELXT - integrated space-group and crystal-structure determination. *Acta Crystallogr., Sect. A: Found.* **2015**, *71*, 3–8. 10.1107/S2053273314026370.

[6] Dolomanov, OV; Bourhis, LJ; Gildea, RJ; Howard, Judith A. K.; Puschmann, Horst. OLEX2 : a complete structure solution, refinement and analysis program. *J. Appl. Cryst.* **2009**, *42*, 339–341. 10.1107/S0021889808042726.
